# Advancing targeted combination chemotherapy in triple negative breast cancer: nucleolin aptamer-mediated controlled drug release

**DOI:** 10.1186/s12967-024-05429-8

**Published:** 2024-07-01

**Authors:** Yuan Ma, Duoli Xie, Zefeng Chen, Xinyang Shen, Xiaoqiu Wu, Feng Ding, Shijian Ding, Yufei Pan, Fangfei Li, Aiping Lu, Ge Zhang

**Affiliations:** 1 Law Sau Fai Institute for Advancing Translational Medicine in Bone and Joint Diseases, School of Chinese Medicine, Hong Kong Baptist University, Kowloon Tsai, Hong Kong SAR 999077 China; 2 Increasepharm & Hong Kong Baptist University Joint Centre for Nucleic Acid Drug Discovery, Hong Kong Science Park, New Territoreis, Hong Kong SAR 999077 China; 3 Department of Obstetrics and Gynecology, Nanfang Hospital, Southern Medical University, Guangzhou, Guangdong 510515 China

**Keywords:** AS1411, Fluorouracil, Paclitaxel, Redox-responsive linker, Triple negative breast cancer, Scheduled drug release

## Abstract

**Background:**

Triple-negative breast cancer (TNBC) is a recurrent, heterogeneous, and invasive form of breast cancer. The treatment of TNBC patients with paclitaxel and fluorouracil in a sequential manner has shown promising outcomes. However, it is challenging to deliver these chemotherapeutic agents sequentially to TNBC tumors. We aim to explore a precision therapy strategy for TNBC through the sequential delivery of paclitaxel and fluorouracil.

**Methods:**

We developed a dual chemo-loaded aptamer with redox-sensitive caged paclitaxel for rapid release and non-cleavable caged fluorouracil for slow release. The binding affinity to the target protein was validated using Enzyme-linked oligonucleotide assays and Surface plasmon resonance assays. The targeting and internalization abilities into tumors were confirmed using Flow cytometry assays and Confocal microscopy assays. The inhibitory effects on TNBC progression were evaluated by pharmacological studies in vitro and in vivo.

**Results:**

Various redox-responsive aptamer-paclitaxel conjugates were synthesized. Among them, AS1411-paclitaxel conjugate with a thioether linker (ASP) exhibited high anti-proliferation ability against TNBC cells, and its targeting ability was further improved through fluorouracil modification. The fluorouracil modified AS1411-paclitaxel conjugate with a thioether linker (FASP) exhibited effective targeting of TNBC cells and significantly improved the inhibitory effects on TNBC progression in vitro and in vivo.

**Conclusions:**

This study successfully developed fluorouracil-modified AS1411-paclitaxel conjugates with a thioether linker for targeted combination chemotherapy in TNBC. These conjugates demonstrated efficient recognition of TNBC cells, enabling targeted delivery and controlled release of paclitaxel and fluorouracil. This approach resulted in synergistic antitumor effects and reduced toxicity in vivo. However, challenges related to stability, immunogenicity, and scalability need to be further investigated for future translational applications.

**Supplementary Information:**

The online version contains supplementary material available at 10.1186/s12967-024-05429-8.

## Introduction

Triple-negative breast cancer (TNBC) accounts for 15%~25% of all breast cancers [1], which is characterized by clinical features such as strong invasiveness, high likelihood of relapse, high metastatic potential, and poor prognosis. TNBC lacks the expression of estrogen receptor (ER), progesterone receptor (PR), and human epidermal growth factor receptor-2 gene (HER2). Due to the abnormal phenotype, TNBC patients cannot benefit from widely used HER2-targeted therapy or hormone therapy [2]. Currently, chemotherapeutics remains the reference treatment of TNBC patients [3]. Paclitaxel (PTX) is a tetracyclic diterpene compound that impedes cell mitosis by tubulin inhibition [4], whereas 5-fluorouracil (5FU) disrupts DNA/RNA synthesis in tumors. Both PTX and 5FU are FDA-approved frontline drugs for breast cancer (BC) treatment [5, 6]. However, both PTX and 5-FU exhibit limited target specificity, leading to systemic side-effects such as myelosuppression, neurotoxicity, and gastrointestinal irritation [7, 8]. Indeed, the use of either PTX or 5FU as monotherapy has demonstrated ineffective outcomes in the treatment of TNBC [6]. Combining drugs with diverse therapeutic mechanisms is a fundamental strategy in tumor chemotherapy [9]. Significant findings from clinical studies have revealed that incorporating capecitabine (5FU prodrug) as adjuvant chemotherapy after PTX-based standard treatment significantly prolonged the progression-free survival (DFS) of TNBC patients [10–13]. It’s worth noting that there is a fixed sequence of administration for PTX and 5FU: PTX must be administered before initiating long-term administration of 5FU [14–16]. Interestingly, the administration of PTX prior to 5FU showed a synergistic effect, because PTX could enhance the sensitivity of tumor cells to 5FU by reducing thymidylate synthase expression [17]. Conversely, the administration of 5FU prior to PTX showed an antagonistic effect, possibly by preventing tumor cells from entering G_2_-M phase [18]. Nevertheless, due to the substantial difference in pharmacokinetic performance between PTX and 5FU in vivo [19, 20], it is challenging to precisely co-deliver PTX and 5FU to tumor tissue, and then to perform scheduled drug release (PTX prior to 5FU) in the clinic [21].

In recent years, attention is shifting from conventional drug to targeted drug in the realm of cancer therapy. Researchers have been exploring new targeted therapies that that focus on different molecules overexpressed in TNBC. In 2021, U.S. Food and Drug Administration (FDA) approved the first antibody (Trop2-targeting)-drug (SN38) conjugate (named sacituzumab govitecan) for the treatment of TNBC patients who have previously received at least 2 chemotherapies with advanced disease [22]. However, approximately 70% TNBC patients do not respond to sacituzumab govitecan treatment [23]. On the one hand, heavily modified antibody embodies the risk of difficult transmembrane, strong immunogenicity, rigid storage requirements, reduced target affinity, altered pharmacokinetics and increased heterogeneity [24, 25]. On the other hand, the pH-responsive carbonate linker (named CL2A) in sacituzumab govitecan was not sufficiently stable in plasma [26, 27], restricting its pharmacokinetics and pharmacodynamics properties. Thus, it is crucial to develop more targeted options for precise TNBC treatment.

Aptamers as targeting-components are a promising modality for cancer treatment, conferring further advantages such as fast screening, rapid cell penetration, low immunogenicity as well as easy synthesis, modification and industrialization [28, 29]. In approximately 80% of TNBC cases, nucleolin has been reported to be overexpressed [30, 31]. The highly expressed nucleolin is related to TNBC metastasis and tumor relapse [32], which is an attractive target for TNBC treatment [33]. ACT-GRO-777 (also known as AS1411) is a nucleolin-targeting aptamer entering clinical phase II study (NCT00740441) [34]. AS1411 could mediate the conjugated cargo internalization into TNBC cells through clathrin-dependent endocytosis [35], and exhibit a slow degradation kinetic to release guanine-based degradation products [36]. Thus, AS1411 conjugated fluorouracil by a non-cleavable linker, such as a phosphodiester bond, could significantly enhance the tumor-targeting ability, and then perform a slow release of fluorouracil.

Since the antitumor efficacy of PTX and 5FU is highly schedule-dependent, the scheduled release of PTX and 5FU is critical for effective TNBC treatment. Redox species significantly contribute to the development of tumor microenvironment (TME)-activating prodrugs [37]. On the one hand, the concentration of glutathione (GSH) in cancer cells can reach 2–10 mM, which is 7–10 times greater than that found in normal tissues [38, 39]. On the other hand, the concentration of H_2_O_2_ in cancer cells can reach 5–1000 µM, which is also much higher than that in normal cells (0.001-0.7 µM) [40]. However, the antitumor efficacy of redox-responsive prodrug with single reduction-responsivity may be restricted by heterogeneous redox microenvironment in tumors. H_2_O_2_ and GSH dual-activated prodrugs enable to address the tumor heterogeneity concerns [41]. Thus, AS1411 conjugated paclitaxel by a redox-dual stimuli cleavable linker, such as a thioether bond, could significantly enhance the tumor-targeting ability, and then perform a rapid release of paclitaxel.

In the study, we designed and synthesized various redox-responsive floxuridine modified AS1411-paclitaxel conjugates with the aim of selective delivery and scheduled release of paclitaxel and 5FU in TNBC. Pharmacologically, fluorouracil modified AS1411-PTX conjugate with a thioether linker (FASP) significantly improved antitumor activity and reduced toxicity in vitro and in vivo. Mechanistically, fluorouracil modification at site 6 facilitated the modified AS1411 enhancing its binding ability for higher specificity. Upon nucleolin-mediated endocytosis, the paclitaxel and fluorouracil performed scheduled drug release and combination antitumor effects. Our findings provided new possibilities for the development of TNBC targeted combination chemotherapy.

## Materials and methods

### Materials

The single-stranded DNAs were purchased from BIOSYNTECH. The chemical reagents were purchased from Aldrich Sigma. The Fetal Bovine Serum (FBS) and Accutase were purchased from Thermo Fisher Scientific. The Phosphate-Buffered Saline (PBS) was purchased from HAKATA. The Dulbecco’s modified Eagle’s medium (DMEM) was purchased from Omacgene. The nuclease-free water (M9011) was purchased from Magic-bio. The 0.25% Trypsin-EDTA (211,042) was purchased from NEST Biotechnology Co. Ltd. (Wuxi, China). The 1.5 mL microcentrifuge tubes (123,201) were purchased from SORFA. The Annexin V-FITC/PI Apoptosis Kit was purchased from Beyotime. The Cell Counting Kit-8 kit (C6005) was purchased from NCM Biotech. The serum-free cell freezing medium (HC01) was purchased from Beijing T&L Biological Technology Co., Ltd. The cells were purchased from ATCC.

### Synthesis of aptamer-paclitaxel conjugates

The AS1411-PTX conjugates were synthesized as described in supporting information. Briefly, redox-responsive di-acids were initially synthesized. These di-acids were then linked to PTX, resulting in the formation of carboxylic acid derivatives. The obtained carboxylic acid derivatives were subsequently conjugated to amino-DNA. This conjugation process led to the generation of various types of aptamer-paclitaxel conjugates (ApDCs).

### CCK-8 assay for cell viability

Cell viability was assessed using the Cell Counting Kit-8 kit. Briefly, MDA-MB-231 cells, 4T1 cells, and MIHA cells were seeded in 96-well plates at a density of 5,000 cells per well and incubated overnight for adherence. Solutions of ApDCs and controls were prepared in medium, ranging in concentration from 0.12 nM to 500 nM. After removal of the cell culture medium, the solutions of ApDCs and controls were added, followed by a 72-hour incubation at 37 °C. After the incubation period, 100 µL of culture medium containing 10% CCK-8 solution was added to each well. The plates were further incubated for 2 h and then read at 450 nm using a microplate reader.

### Drug release assay

ApDCs (0.15 nmol) were dissolved in 0.1×phosphate buffer saline (PBS, 60 µL), Dithiothreitol solution (DTT, 10 mM, 60 µL) and Hydrogen peroxide (H_2_O_2_, 1 mM, 60 µL), respectively. The mixture was incubated at 37 °C. At timed intervals (0 h ~ 24 h), the intact ApDCs were analyzed utilizing an Agilent 1290 HPLC system equipped with a UV detector set at 260 nm. The HPLC method employed a YMC-Triart C18 column (3 μm, 150 mm × 4.6 mm) with a mobile phase consisting of phase A (Acetonitrile, ACN) and phase B (Triethylammonium acetate, TEAA, 50 mM). A gradient elution was run from 2 to 55% of phase A in 30 min at a flow rate of 0.5 mL·min^− 1^ with ambient column temperature. The percentage of intact ApDCs was normalized with the initial amount treated as 100%.

### Cellular apoptosis assay

Cellular apoptosis was assessed using the Annexin V-FITC/PI Apoptosis Kit. Briefly, MDA-MB-231 cells were seeded in 6-well plates at a density of 4 × 10^5^ cells per well and incubated overnight for adherence. Solutions of ApDCs and controls were prepared in medium at 200 nM. After removal of the cell culture medium, the solutions of ApDCs and controls were added, followed by a 48-hour incubation at 37 °C. After the incubation period, cells were harvested using 0.25% Trypsin-EDTA, rinsed using cold PBS, and resuspended in 200 µL 1×Annexin-V binding buffer. Next, 5 µL Annexin-V-FITC (fluorescein isothiocyanate) and 10 µL PI (propidium iodide) were added to the cell suspension and incubated at room temperature for 15 min in the dark with gentle vertexing. Quantitative determination was performed using a flow cytometer. By analyzing the fluorescence signals obtained from the flow cytometer, we were able to assess the proportion of cells undergoing early apoptosis (Annexin-V positive, PI negative), late apoptosis (Annexin-V positive, PI positive), and necrosis (PI positive) in response to the treatment with ApDCs and respective controls.

### ELONA assay for relative binding ability

The relative binding ability of modified aptamers to sclerostin was evaluated using enzyme-linked oligonucleotide assays (ELONA). To perform the assay, a 96-well microtiter plate was coated with 160 ng of nucleolin protein in SELEX B&W buffer (1 mM MgCl_2_ and 0.05% Tween 20 in 1×PBS, 100 µL) and incubated overnight at 4 °C. The plate was then blocked with blocking buffer (0.1% Tween 20 and 1% BSA in 1×PBS) for 1 h at room temperature, followed by washing with SELEX B&W buffer four times. Biotinylated aptamers (1 µM) in SELEX B&W buffer (100 µL) were added to each well and incubated for 45 min at room temperature with gentle shaking. After binding, the plate was washed four times with SELEX B&W buffer to remove non-specific and weakly bound molecules. Next, 0.01% streptavidin-HRP (100 µL) was added to each well, followed by a 30-min incubation and washing with washing buffer (1 mM MgCl_2_, 0.1% Tween 20, and 0.1% BSA in 1×PBS, 100 µL) for four times. TMB substrate (50 µL) was added to each well and incubated for 20 min, after which the reaction was stopped by adding 2 M H_2_SO_4_ (50 µL). The absorbance at 450 nm was measured using a microplate reader. The absorbance of the modified AS1411 was normalized to the unmodified AS1411 treated as 100%.

### SPR assay for binding affinity

The binding affinity of modified aptamers to sclerostin was determined using surface plasmon resonance (SPR) assay. Initially, the nucleolin protein was immobilized onto a CM5 chip following the manufacturer’s protocol. The chip surface was activated using a mixture of 0.5 M N-hydroxysuccinimide (NHS) and 0.1 M 1-ethyl-3-(3-dimethylaminopropyl) carbodiimide hydrochloride (EDC) in a 1:1 volume ratio. Then, nucleolin diluted in 10 mM sodium acetate (pH = 5.0) was added to the activated surface. For the interaction analysis between ApDCs and nucleolin, ApDCs were diluted in 1×PBS at concentrations ranging from 0.156 µM to 10 µM. To ensure consistent binding signals, seven consecutive injections of equal volumes were performed. After each injection, the chip surface was regenerated using 50 mM sodium hydroxide (NaOH). The dissociation constant (K_d_) was determined using a GE Biacore X100 SPR System.

### Confocal microscopy assay for cellular endocytosis

MDA-MB-231 cells were seeded in a 24-well plate at a density of 2 × 10^5^ cells per well and incubate overnight. Then, the cells were incubated with Cy5-labeled AS1411, FA, ASP and FASP, each at a concentration of 250 nM. Subsequently, the cells were incubated at 37 °C for 4 h to allow for internalization of the aptamers. In the last 15 min of the incubation period, a volume of 2 µg·mL^− 1^ Hoechst 33,342 (blue) was added to the cells. After the incubation, the cells were gently washed to remove any unbound or extracellular aptamers. Finally, the cells were visualized using confocal microscopy.

### FCM assay for cellular uptake

MDA-MB-231 cells and MCF10A cells were seeded in a 10 cm dish at a density of 2.0 × 10^5^ cells per dish and incubated overnight. The cells were then washed three times with PBS and detached using Accutase. Subsequently, the cells were treated with Cy5-labeled AS1411, FAS1411, ASP, and FASP at a concentration of 50 nM for 3 h. After the treatment period, the cellular uptake levels of FASP and the control groups were analyzed using flow cytometry (FCM) in the APC channel.

### Animal study for antitumor efficacy in vivo

The Laboratory Animal House of Hong Kong Baptist University provided housing for the mice used in this study. The animal facility maintained a regulated environment with controlled temperature and a 12 h light/dark cycle, while food and water were freely accessible to the mice throughout the study. Prior to conducting any experiments, a minimum of one week was allocated for the mice to acclimate to their new environment. All in vivo studies were conducted in compliance with ethical guidelines and received approval from the Animal Experimentation Ethics Committee of the Hong Kong Baptist University (REC/22–23/0401).

Eight-week-old female BALB/c nude mice were inoculated subcutaneously with 1 × 10^7^ MDA-MB-231 cells in the armpit. After tumors were observed within 3 weeks, the mice were randomly divided into three groups (seven mice in each group) for further experimentation. The mice were administered with FASP, ASP, PTX, FTSP, FUDR twice a week for three weeks via subcutaneous injection at a dosage of 1.5 µmol·kg^− 1^, and the vehicle group was administered with equivolume of PBS. Tumor size and body weight were measured twice a week, with intervals of 3–4 days. At the end of the treatment, the mice were euthanized, and the tumors were weighted. The blood samples were obtained for biochemical analyses.

### Animal study for biodistribution effect in vivo

The biodistribution studies were performed in MDA-MB-231 inoculated BALB/c nude mice. After tumors were observed within 3 weeks, the mice were randomly divided into two groups for further experimentation. The mice were administered with Cy5 labeled FASP and FTSP via subcutaneous injection at a dosage of 0.5 µmol·kg^− 1^. After 4 h, the mice were euthanized. The heart, liver, spleen, lung, kidney and tumor were obtained for images utilizing Imaging Station Maestro 2 (CRI, MA, USA).

### CD assay for secondary structures

The secondary structures of PTX, AS1411, FA and FT were analyzed using a J-715 circular dichroism (CD) spectropolarimeter. The background signal of the binding buffer was measured and subtracted from the CD spectrum. Before the assay, the chamber was deoxygenated with dry purified nitrogen (99.99%) and kept in the nitrogen atmosphere during experiments. At wavelengths from 320 to 220 nm, CD spectra were collected at 0.1 nm intervals by accumulation of two scans at 20 nm·min^− 1^ with a 1 nm bandwidth and time constant of 4 s.

### Structural preparation of aptamers

The initial structure of AS1411 was obtained by using the Z-G4 with d[T(GGT)4TG(TGG)3TGTT] (PDB ID: 4U5M) as a template for modification. The AS1411 structure was derived by mutating the penultimate base thymine (T) to guanine (G) and deleting the thymine bases at both ends using Discovery Studio. Additionally, the methyl group on the carbon atom at position 5 of the T6 base in the initial AS1411 structure was mutated to a fluorine atom, resulting in the initial structure of FA. The coordinates of the human NCL RBD1,2 (PDB ID: 2KRR) were obtained from the RCSB Protein Data Bank (RCSB PDB) (https://www.rcsb.org/), specifically selecting the ninth model as the initial human NCL RBD1,2 structure based on previous report [42].

### Molecular docking

The HDOCK server (http://huanglab.phys.hust.edu.cn/software/hdocklite/) (10.1038/s41596-020-0312-x) was used for molecular docking. Rigid-body docking was performed for each pair of receptor and ligand, and the server provided the top 10 predicted docking results for visualization. The initial Protein-Aptamer complex structure for subsequent molecular dynamics simulations was selected based on the model with the highest docking score.

### Molecular dynamics simulation

Molecular dynamics simulations were conducted for the complexes of the aptamer and NCL RBD1/2 using Discovery Studio. The CHARMm force field was applied to the complexes using the Prepare Protein module of Discovery Studio. The complexes were solvated in a cube box containing water molecules, with a minimum distance of 12 Å between the solute and the box border. Potassium or chloride ions were added to neutralize the system to a concentration of 150 mM, which has been validated for the folding of AS1411. The system underwent minimization, temperature equilibration, and a 15 ns production run. Trajectory analysis was performed using Discovery Studio, and the binding conformation with the lowest total energy was selected. The binding free energy of this conformation was calculated, and salt bridge analysis was conducted using the online tool PLIP and PyMOL for visualization. The simulation results were analyzed for Root Mean Square Deviation (RMSD) and changes in energy.

### Statistical analysis

All variables were expressed as mean ± standard deviation. One-way ANOVA with Tukey’s post-hoc test was performed to determine the inter-group differences in the study variables. All the statistical data were analyzed by GraphPad Prism, and *P* < 0.05 was considered to be statistically significant. For the in vivo experiments, the animals were grouped randomly and blindly to researchers.

## Results

### Different redox-responsive aptamer-paclitaxel conjugates were synthesized

The synthesis of compound 4/5 commenced with compound 1 and 1,4-oxathiane-2,6-dione to afford compound 2, accompanied by moderate pyridine (Py) as an activator. Compound 3 was then prepared through treating compound 2 with N, N’-dicyclohexylcarbodiimide (DCC) and N-hydroxysuccinimide (NHS). Without further purification, the activated carboxyl group in compound 3 reacted with 3′-amino DNAs [AS1411 or Negative control (NC)] in the present of NaHCO_3_ (pH = 8.35), yielding crude compound 4 (Scheme S1). Additionally, compound 6 was constructed by oxidizing propanethiol in the present of iodine (I_2_) and triethylamine (TEA), followed by refluxing in acetyl chloride to obtain compound 7. After reacting with paclitaxel, compound 8 was then activated and reacted with 3′-amino DNAs in the phosphate buffer (pH = 8.0) to produce compound 9 (SchemeS2). Furthermore, compound 10 was gained through the reaction of propanethiol and acetone. Then, compound 10 was directly conjugated to PTX, the afforded compound 11 reacted with 3’-amino DNAs in the present of equal amount of DCC, hexafluorophosphate benzotriazole tetramethyl uronium (HBTU), N,N-diisopropylethylamine (DIPEA) to afford compound 12 (Scheme S3). The structures of critical intermediates were confirmed using ^1^H-NMR, ^13^C-NMR, and high-resolution mass spectrometry (Attachment figures). Finally, the products were purified and confirmed by mass spectrometry (Table S1, Attachment figures

### AS1411-paclitaxel conjugate with thioether linker exhibited high anti-proliferation ability against TNBC cells and its targeting ability was further improved through fluorouracil modification

**Fig. 1:**
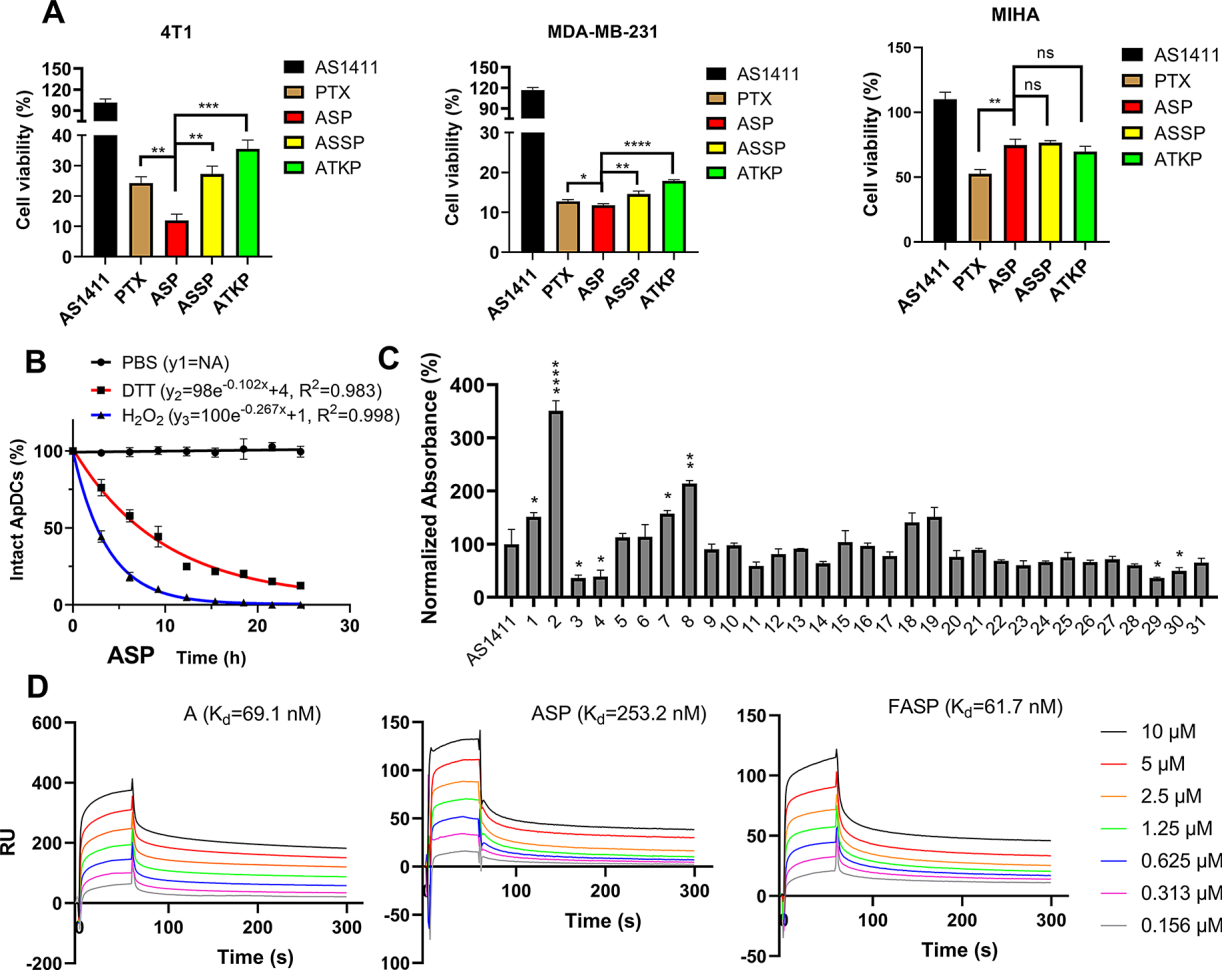
AS1411-paclitaxel conjugate with thioether linker exhibited high anti-proliferation ability against triple negative breast cancer cells and its targeting ability was further improved through fluorouracil modification. (**A**) Anti-proliferation effect of AS1411-paclitaxel conjugates with different redox-responsive linkers on 4T1 cells, MDA-MB-231 cells and MIHA cells. The concentration of AS1411-paclitaxel conjugates was 250 nM. Data were expressed as mean ± standard deviation from at least three replicates, followed by one-way ANOVA with Tukey’s post-hoc test. ns *P* > 0.05; * *P* < 0.05; ** *P* < 0.01; *** *P* < 0.001; **** *P* < 0.0001 versus PTX group. (**B**) Drug release of AS1411-paclitaxel conjugates in phosphate buffered saline (PBS, 0.1×), reduction microenvironment (DTT, 10 mM), oxidation microenvironment (H_2_O_2_, 1 mM), respectively. ApDCs (0.15 nmol) were incubated in different buffers at 37 °C and sampled every three hours. The normalized intact ApDCs at 0 h was treated as 100%. Data were presented as mean ± standard deviation from three replicates. (**C**) Binding ability of fluorouracil modified AS1411s to nucleolin. The X-axis represents modified AS1411s. The Y-axis was the normalized absorbance at 450 nm, which represented the relative binding ability of the modified aptamer to nucleolin. The absorbance of unmodified AS1411 was treated as 100%. Data were expressed as mean ± standard deviation from at least three replicates, followed by one-way ANOVA with Tukey’s post-hoc test. * *P* < 0.05; ** *P* < 0.01; **** *P* < 0.0001 versus unmodified AS1411 group. Sequence 2 was fluorouracil modified at site 6 in AS1411 (named FA). (**D**) Binding affinity of fluorouracil modified AS1411-paclitaxel conjugates to nucleolin. A represented AS1411 treatment. ASP represented AS1411-paclitaxel conjugate with thioether linker treatment. ASSP represented AS1411-paclitaxel conjugate with disulfide linker treatment. ATKP represented AS1411-paclitaxel conjugate with thioketal linker treatment. FASP represented fluorouracil modified AS1411-paclitaxel conjugate with thioether linker treatment. RU represented resonance units. The concentrations of the ApDCs and aptamers ranged from 0.156 µM to 10 µM

To evaluate the cytotoxic effects of aptamer-drug conjugates (ApDCs) with different redox-stimuli responsive linkers, cell counting kit-8 (CCK-8) assay was performed on human TNBC cells (MDA-MB-231), mouse TNBC cells (4T1) and normal human liver cells (MIHA), respectively. Our in vitro data demonstrated that AS1411-paclitaxel conjugate with thioether linker (ASP) exhibited higher anti-proliferation ability against TNBC cells compared to that with either disulfide linker (ASSP) or with thioketal (ATKP) linker. Additionally, all AS1411-paclitaxel conjugates showed reduced toxicity against normal liver cells (Fig. 1A). It indicated that AS1411-paclitaxel conjugate with thioether linker could improve anti-TNBC activity and reduce toxicity in vitro. It was worth noting that ASP could be effectively endocytosed into MDA-MB-231 cells, while NC sequence-paclitaxel conjugate with thioether linker (NCSP) could be rarely endocytosed into MDA-MB-231 cells (Figure S1). It indicated that aptamer played an important role on cellular trafficking of paclitaxel. To validate the oxidation-reduction dual-activated drug release, high performance liquid chromatography (HPLC) assay was performed to measure the relative intact ApDCs after incubation in physiological buffer (PBS), reduction buffer (DTT) or alternative oxidation buffer (H_2_O_2_). Our in vitro data demonstrated that ASP sustained high stability after incubation in physiological buffer at 37 °C for 24 h. Notably, ASP could be cleaved either in reduction buffer or in oxidation buffer within pointed time (Fig. 1B), suggesting in a rapid release of paclitaxel. However, our surface plasmon resonance (SPR) data showed that the binding affinity of ASP to nucleolin (K_d_=253.2 nM) was 3.7 folds lower than that of AS1411 (K_d_=69.1 nM) (Fig. 1D). It could be explained by the fact that the steric hindrance of PTX impacted the interactions between aptamer to target protein.

To reverse the suppressive effect of PTX on AS1411 in binding affinity, post-SELEX modification strategies were conduct for AS1411 affinity optimization, uridine (U), 5-indole-uridine (Indole-U) and 5-fluoro-uridine (5FU) modifications were introduced into AS1411, individually (Table S2). Structure-activity relationship investigations by enzyme-linked oligonucleotide assay (ELONA) showed that deletion of 5-methy in thymidine (i.e. U) significantly reduced the binding affinity of modified AS1411, indicating the vital roles of 5-methy in thymidine. Furthermore, replacement of 5-methy to hydrophobic 5-indole (i.e. Indole-U) also reduced the binding affinity of modified AS1411, indicating the large steric hindrance group would impact their interactions. Noteworthily, replacement of 5-methy to 5-fluorine (i.e. 5FU) significantly improved the binding affinity of modified AS1411, especially in site 6 (Figure S2). Importantly, a single 5FU modification could facilitate the highest binding affinity of the modified AS1411, whereas multiple 5FU modification would mitigate the binding affinity of the modified AS1411 (Fig. 1C). To validate the high affinity effect of 5FU in aptamer, the binding affinity of 5FU modified AS1411 (FA), 5FU modified AS1411-paclitaxel conjugate with thioether linker (FASP), negative control sequence (T), 5FU modified negative control sequence (FT), negative control sequence-paclitaxel conjugate with thioether linker (TSP) were determined by SPR assay. It was found that the binding affinity of FA (K_d_=24.8 nM) was improved for 2.8 folds. There is no significant difference in binding affinity parameter between AS1411 (K_d_=69.1 nM) and FASP (K_d_=61.7 nM) (Fig. 1D, S2). It indicated that 5FU modification indeed reverse the suppressive effect of PTX on AS1411 in binding affinity. In contrast, either T, FT or TSP showed no binding affinity to nucleolin (Figure S3). It suggested that the conjugated 5FU and PTX would not cause nonspecific binding.

### Fluorouracil modified AS1411-paclitaxel conjugate with thioether linker could target to TNBC cells, resulted in significantly improved anticancer activity in vitro

**Fig. 2:**
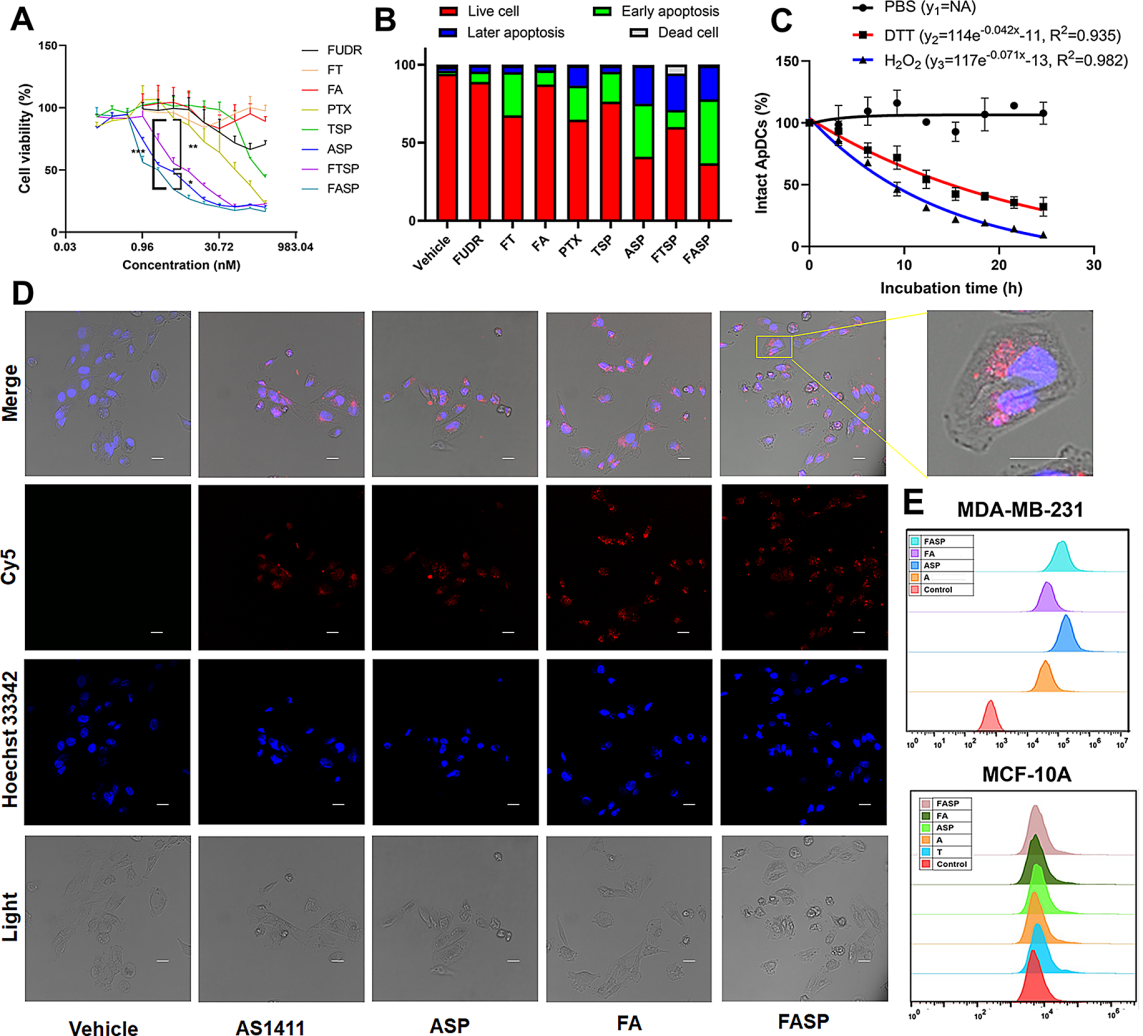
Fluorouracil modified AS1411-paclitaxel conjugate with thioether linker could target to MDA-MB-231 cells, resulted in significantly improved anticancer activity in vitro. (**A**) Anti-proliferation effect of FUDR, FT, FA, PTX, TSP, ASP, FTSP and FASP on MDA-MB-231 cells. The concentrations of each sample ranged from 0.12 nM to 500 nM. Data were expressed as mean ± standard deviation from at least three replicates, followed by one-way ANOVA with Tukey’s post-hoc test. * *P* < 0.05; ** *P* < 0.01; *** *P* < 0.001. (**B**) The apoptosis of MDA-MB-231 cells after 72 h treatment of Vehicle, FUDR, FT, FA, PTX, TSP, ASP, FTSP and FASP. The concentration of each sample was 200 nM. The percentage of live cells, early apoptosis, later apoptosis, and dead cells were analyzed by flow cytometry using TransDetect^®^ Annexin V-FITC/PI Cell Apoptosis Detection Kit. (**C**) Drug release of fluorouracil modified AS1411-paclitaxel conjugates (0.15 nmol) in phosphate buffered saline (PBS, 0.1×), reduction microenvironment (DTT, 10 mM), oxidation microenvironment (H_2_O_2_, 1 mM), respectively. Data were presented as mean ± standard deviation from three replicates. (**D**) The cellular endocytosis of Vehicle, AS1411, ASP, FA and FASP in MDA-MB-231 cells were evaluated by confocal microscopy. The concentration of each sample was 50 nM. The representative images showed the cells (light). The AS1411, ASP, FA and FASP were visualized by Cy5 fluorescein (red). The nucleus was stained with Hoechst 33,342 (blue). Scale bar: 20 μm. (**E**) The cellular uptake of AS1411, ASP, FA and FASP in MDA-MB-231 cells evaluated by flow cytometry using APC channel. The concentration of each sample was 50 nM. Control represents no treatment; FUDR represents fluorouracil prodrug treatment; FT represents fluorouracil modified at site 6 in negative control aptamer treatment; TSP represents negative control aptamer-paclitaxel conjugate with thioether linker treatment; FTSP fluorouracil modified negative control aptamer-paclitaxel conjugate with thioether linker treatment. Vehicle represents PBS treatment

After confirming the high binding affinity of fluorouracil modified AS1411-paclitaxel conjugate with thioether linker (FASP), the anti-proliferative ability of floxuridine (FUDR, 5FU prodrug), FT, FA, PTX, TSP, ASP, FTSP and FASP was performed on several types of nucleolin-positive human TNBC cells (MDA-MB-231 cells, MDA-MB-453 cells and BT-20 cells) (Figure S4A-B). Our in vitro data demonstrated that FTSP group exhibited higher anti-proliferative ability compared to PTX group, indicating a superimposed effect of 5FU and PTX against TNBC cells. Additionally, ASP group exhibited higher anti-proliferative ability compared to PTX group, indicating that AS1411 could facilitate the tumor-targeting ability of PTX. Furthermore, FASP group showed the highest anti-proliferative ability among all groups, which was much higher than either ASP group or FTSP group (Fig. 2A, Figure S4C-D). Consistently, FASP group characterized superior apoptosis induction compared to either FUDR group or PTX group in vitro (Fig. 2B, Figure S4E-F). Collectively, it indicated that AS1411 significantly enhanced the tumor-targeting ability of the conjugated paclitaxel and fluorouracil in TNBC cells, resulting in improved antitumor activity. To validate the oxidation-reduction dual-activated drug release, HPLC assay was performed to measure the relative intact ApDCs after incubation in PBS, DTT or H_2_O_2_ buffers (Fig. 2C). Our in vitro data demonstrated that FASP sustained high stability after incubation in physiological buffer at 37 °C for 24 h. Notably, FASP could be cleaved either in reduction buffer or in oxidation buffer within hours, suggesting that PTX could be rapid released in redox tumor microenvironment.

To enable targeted drug delivery, we conducted further investigations to assess the cellular uptake ability of AS1411, ASP, FA, and FASP using confocal microscopy and flow cytometry. To visualize their uptake in cells, we labeled the aptamers with Cy5 fluorescence (red) and stained the nucleus with Hoechst 33,342. The confocal microscopy images revealed a robust Cy5 fluorescence signal surrounding the nucleus in MDA-MB-231 cells treated with the FASP group. Conversely, no Cy5 fluorescence was detected in MDA-MB-231 cells treated with the vehicle group (Fig. 2D). Consistently, the flow cytometry images revealed a strong Cy5 fluorescence signal in MDA-MB-231 cells treated with the FASP group. However, neither the TNBC cells (MDA-MB-231 cells) treated with the vehicle group nor the normal breast cells (MCF10A cells) treated with the FASP group exhibited any Cy5 fluorescence signal (Fig. 2E). Collectively, these findings indicated that the cellular uptake of FASP occurred through a target cell-specific ligation approach.

### Fluorouracil modified AS1411-paclitaxel conjugate with thioether linker could accumulate in tumors, resulting in significantly improved anticancer activity in vitro

**Fig. 3:**
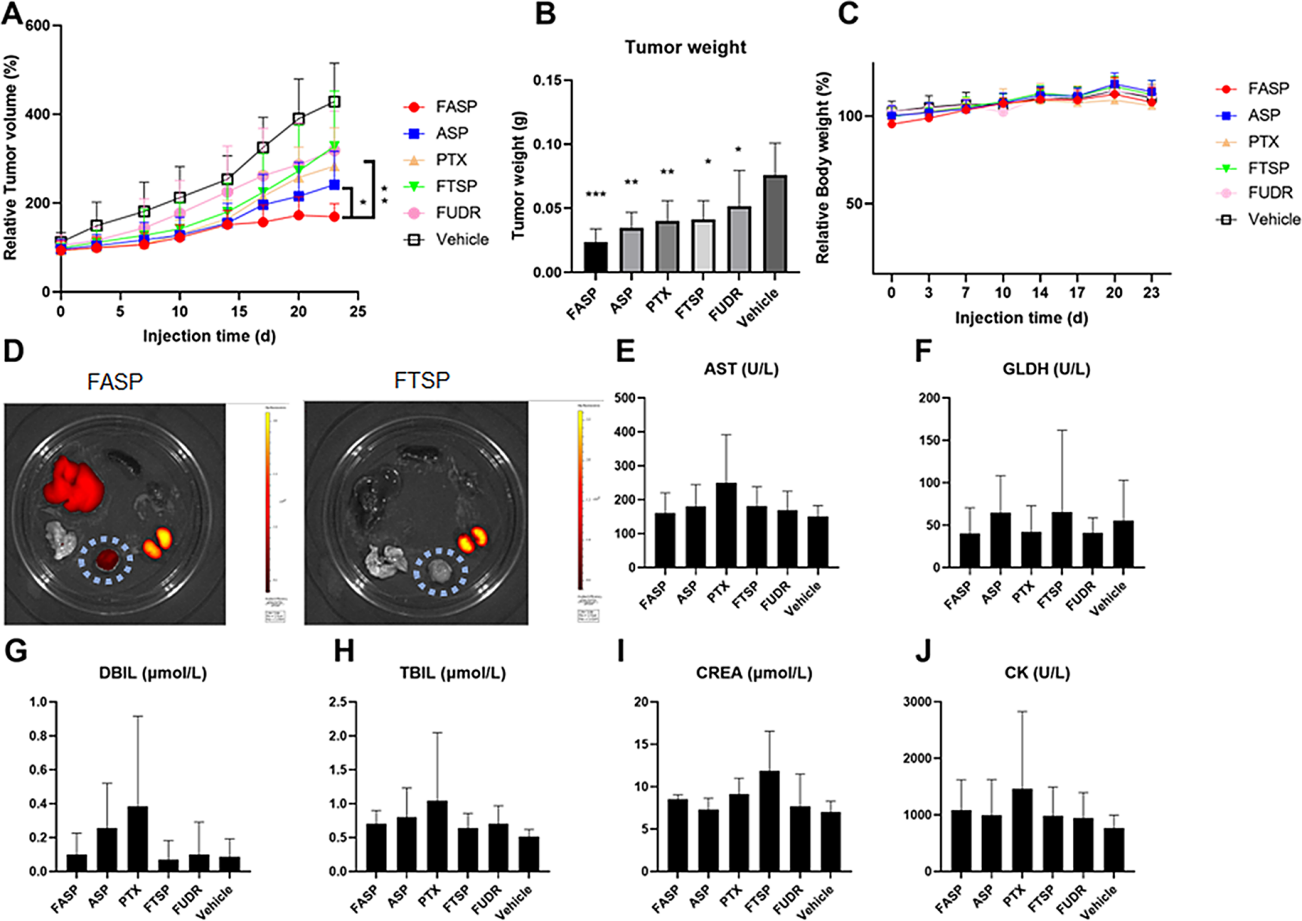
Fluorouracil modified AS1411-paclitaxel conjugate with thioether linker could accumulate in tumors, resulting in significantly improved anticancer activity in vivo. (**A**) The relative tumor volume of the MDA-MB-231 xenografted tumor treated with FASP, ASP, PTX, FTSP, FUDR and Vehicle (30 nmol, twice per week), respectively. The tumor volumes were the normalized to the mean tumor volume at day 0. (**B**) The tumor weights of the xenografted tumor after 23 days of different treatments from the groups indicated. (**C**) The relative body weight of the MDA-MB-231 inoculated mice of different treatments from the groups indicated. The body weights were the normalized to the mean body weight at day 0. (**D**) The distribution of MDA-MB-231 inoculated mice of different treatments from FASP group and FTSP group after 4 h. The groups indicated were visualized by Cy5 fluorescein. After mice sacrificed, the tissues, including heart, liver, spleen, lung, kidney, and tumor were isolated and photographed. (**E-J**) The kidney and liver functions of the MDA-MB-231 inoculated mice of different treatments from the groups indicated. *Notes*: AST indicated aspartate aminotransferase, DBIL indicated direct bilirubin, TBIL indicated total bilirubin, CREA indicated creatinine, CK indicated creatine kinase, GLDH indicated glutamate dehydrogenase. Data were expressed as mean ± standard deviation from seven replicates (*n* = 7), followed by one-way ANOVA with Tukey’s post-hoc test. * *P* < 0.05; ** *P* < 0.01; *** *P* < 0.001 versus Vehicle group. All statistical analyses were performed using GraphPad Prism 8.0.1 software

To evaluate the inhibitory effect of FASP on tumor growth in vivo, the preliminary pharmacodynamics, safety and distribution studies were conducted to measure the tumor size, tumor weight, body weight, kidney and liver function parameters, drug distribution in MDA-MB-231 inoculated Balb/c-nu mice. The mice were subcutaneously administrated (twice per week, 3.5 weeks) with FASP, ASP, PTX, FTSP, FUDR and Vehicle. For tumor volume and tumor weight (Fig. 3A-B), FASP group exhibited higher antitumor growth ability compared to PTX group and ASP group, indicating a superimposed effect of 5FU and PTX against TNBC tumor growth in vivo.For body weight, there were no significant differences of different treatments from the groups indicated (Fig. 3C). For drug distribution, FASP group exhibited higher tumor accumulation compared to FTSP group, indicating an enhanced tumor-targeting ability (Fig. 3D). For kidney and liver functions, PTX group or FTSP group modestly increased aspartate aminotransferase (AST), direct bilirubin (DBIL), total bilirubin (TBIL), creatinine (CREA), creatine kinase (CK) and glutamate dehydrogenase (GLDH), whereas FASP group restored the above parameters to wide-type level (Vehicle group) (Fig. 3E-J). It indicated that compared to free chemotherapeutics, FASP group exhibited better safety with reduced toxicity. Collectively, our in vivo data demonstrated that FASP could accumulate in tumors, resulting in significantly improved anticancer activity and reduced toxicity in MDA-MB-231 inoculated mice.

### Insight into the binding mode of fluorouracil modified AS1411 to nucleolin target

**Fig. 4:**
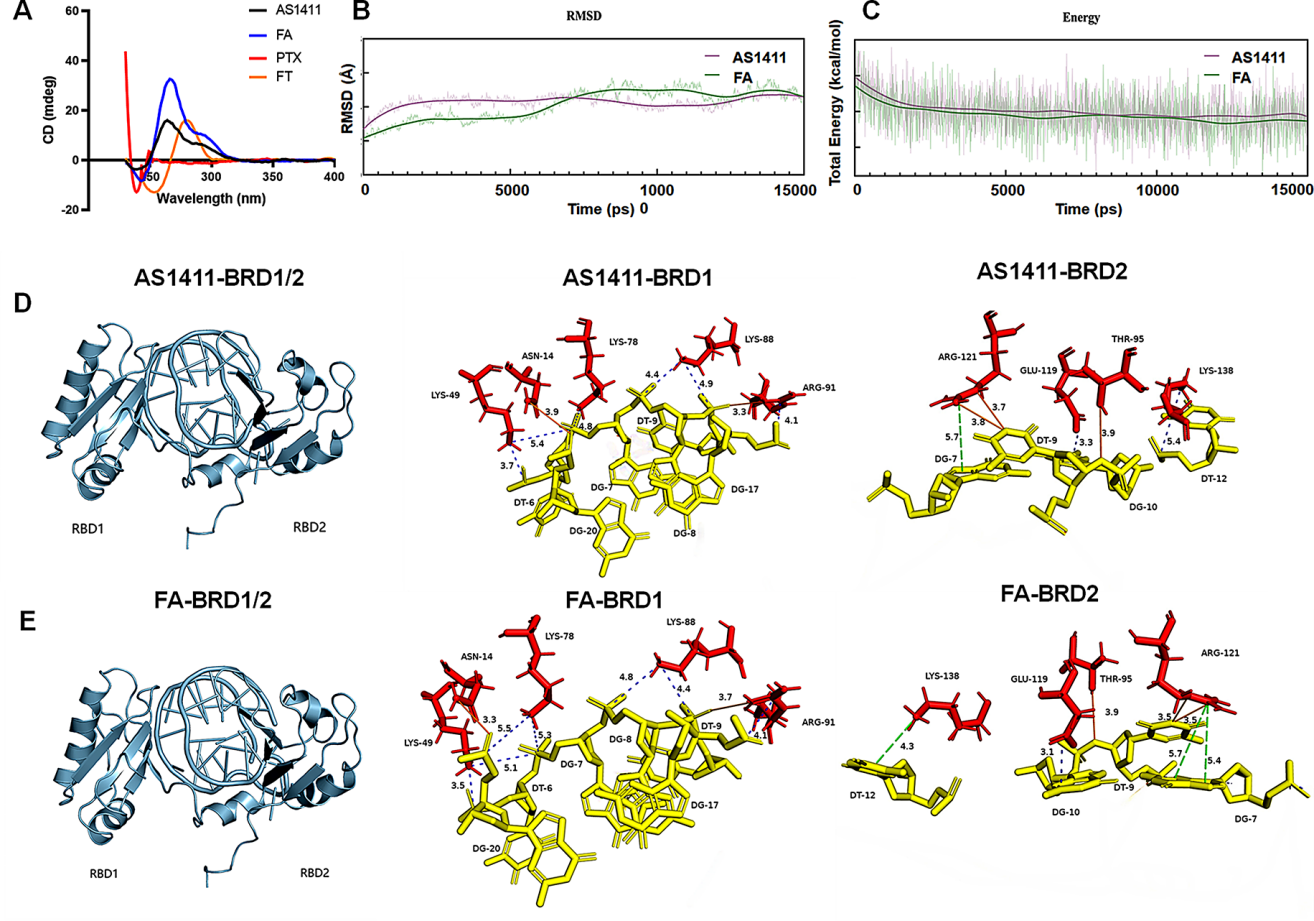
Insight into the binding mode of fluorouracil modified AS1411 to nucleolin target. (**A**) The secondary structures of AS1411, FA, PTX and FT in PBS evaluated by CD spectra. The concentration of each sample was 0.2 mM. (**B**) The RMSD curves of AS1411 and FA evaluated by molecular dynamics simulation. (**C**) The total energy curves of AS1411 and FA evaluated by molecular dynamics simulation. (**D**) The AS1411-NCL BRD complex structure predicted by molecular docking. (**E**) The FA-NCL BRD complex structure predicted by molecular docking

To investigate the specific binding between fluorouracil (FA) and nucleolin, CD spectra, molecular docking, and molecular dynamics (MD) simulations were conducted to predict the binding mode with an atomic-level descriptions of the high affinity interaction. The CD spectra data demonstrated that fluorouracil modification facilitated the formation of G4 structures in the modified aptamer, as indicated by enhanced signals (Fig. 4A). This structural enhancement contributed to the improved binding ability of the aptamer to nucleolin. The MD simulations confirmed the stability of the predicted structures of AS1411 and FA in solution (Fig. 4B), with FA consistently exhibiting lower total energy compared to AS1411, indicating its conformational stability (Fig. 4C).

Structural analysis revealed the crucial role of fluorouracil modification in the binding of the modified aptamer to the nucleolin RBD1/2 domain. This modification facilitated the formation of new binding sites between T9 and Arg121, as well as G20 and Lys78 (Fig. 4D-E, TableS3). As a result, the number of interactions between the aptamer and nucleolin RBD1/2 domain increased from 14 to 16, while the binding energy decreased from − 1494 kcal/mol to -1565 kcal/mol (Table S3). These findings indicate that the fluorouracil-modified AS1411 exhibited superior targeting ability and stronger interactions with nucleolin, making it a promising candidate for nucleolin-specific binding.

## Discussion

Targeted therapies are essential for improving patient outcomes in triple-negative breast cancer (TNBC). Neoadjuvant treatment involving sequential delivery of specific combinations has become the current standard of care, but its implementation poses challenges. In this study, we investigated the anti-proliferative effects of AS1411-paclitaxel conjugates on TNBC cells, focusing on the influence of a redox-linker. We found that the AS1411-paclitaxel conjugate with a thioether linker (ASP) exhibited superior anti-proliferative properties compared to the conjugate with either a disulfide linker (ASSP) or a thioketal linker (ATKP). This could be attributed to the oxidant/reductant dual-activated characteristics of the thioether linker, leading to a rapid release of caged paclitaxel [43]. Our designed ASP showed improved properties compared to the previously reported oleic acid (OA)-paclitaxel conjugate connected by a thioether linker. However, our SPR data revealed that the binding affinity of ASP (K_d_=253.2 nM) to nucleolin was moderately lower than that of free AS1411 (K_d_=69.1 nM), indicating a steric hindrance effect of the paclitaxel conjugate.

For the sequential delivery of neoadjuvant combinations, we chose fluorouracil as a typical nucleoside drug that could be easily incorporated into AS1411 using a solid-phase synthesis approach. The rapid release of paclitaxel by the cleavable thioether linker and slow release of fluorouracil by the non-cleavable phosphodiester linker allowed for the sequential delivery of paclitaxel and fluorouracil, which has demonstrated effective and feasible results in clinical studies with synergistic effects [44]. In comparison to the reported lipid-encapsulated hollow mesoporous silica system for controlled release of paclitaxel and fluorouracil, our developed fluorouracil-modified AS1411-paclitaxel conjugate with a thioether linker (FASP) is a single-molecule entity with excellent quality control and reliability [21]. To address the reduced binding affinity of the aptamer-drug conjugate, the modification sites of fluorouracil in AS1411 were further investigated. We found that fluorouracil at site 6 facilitated the modified AS1411 enhancing its binding affinity to nucleolin (K_d_=24.8 nM), thereby restoring the binding affinity of FASP (K_d_=61.7 nM) to the same level as free AS1411 (K_d_=69.1 nM).

It has been reported that antimetabolic drugs could facilitate chemotherapy drugs enhancing therapeutic effects and reducing treatment toxicity [45]. Our in vitro data demonstrated that FASP group exhibited higher anti-proliferative ability compared to ASP group, indicating a superimposed effect of 5FU and PTX against TNBC cells. Notably, FASP group could accumulate in tumors, and significantly inhibited the tumor growth in MDA-MB-231 inoculated mouse models. These findings offer new possibilities for developing targeted combination chemotherapy in TNBC. However, aptamer-based drug delivery systems also present potential limitations and challenges for implementation. One significant concern is stability, as aptamers can be prone to degradation under certain physiological conditions. Strategies to improve aptamer stability, such as chemical modifications or encapsulation within protective carriers, need to be explored to ensure effective drug delivery. Immunogenicity is another important consideration. Aptamers, being synthetic nucleic acids, may trigger an immune response in vivo, potentially leading to reduced therapeutic efficacy or unwanted immune reactions. Techniques like sequence optimization or modification can help mitigate immunogenicity risks, but comprehensive evaluation and validation are necessary to ensure their clinical safety and effectiveness. The selection of chemotherapeutics is a limitation. The effective combinations of chemotherapeutics for breast cancer are Carboplatin and Paclitaxel, as well as Epirubicin and Cyclophosphamide. However, we encountered compatibility issues between Carboplatin/Cyclophosphamide and DNA aptamer due to their covalent interactions. As a result, we were unable to combine Epirubicin and Cyclophosphamide using this delivery system. Scalability for clinical translation is a critical challenge. The large-scale production of aptamers can be technically demanding and costly. Methods for efficient and cost-effective synthesis, purification, and characterization need to be developed to enable widespread clinical use.

## Conclusions

In summary, we simultaneously incorporated fluorouracil and paclitaxel into a nulceolin aptamer by a non-cleavable linker (phosphodiester bond) and a redox-stimuli cleavable linker (thioether bond), respectively. Our established fluorouracil modified AS1411-paclitaxel conjugates with thioether linker exhibited efficient recognition of TNBC cells, enabling targeted delivery and scheduled release of PTX and 5FU, resulting in superimposed antitumor effects and reduced toxicity in vivo. Our findings provided new possibilities for the development of TNBC targeted combination chemotherapy. However, challenges related to stability, immunogenicity, and scalability need to be further investigated for future translational applications.

### Electronic supplementary material

Below is the link to the electronic supplementary material.

Supplementary Material 1

## Data Availability

All data included in this study are available upon request by contact with the corresponding authors.
