# Supplementary material for: Advancing targeted combination chemotherapy in triple negative breast cancer: nucleolin aptamer-mediated controlled drug release
Source: J Transl Med. 2024 Jul 1;22:604. doi: 10.1186/s12967-024-05429-8 (PMC11218354; doi:10.1186/s12967-024-05429-8)
Supplement: Supplementary file 1 — Supplementary Material 1 [file 12967_2024_5429_MOESM1_ESM.docx]

**1. Synthesis**

**Scheme S1.** Preparation of thioether bridged AS1411-PTX conjugates. Note for reagents and conditions: (i) 1,4-oxathiane-2,6-dione, Py, THF; (ii) NHS, Py, THF; (iii) Amino-DNA, NaHCO_3_, H_2_O, DMF. Compound 4 indicated ASP.

**Scheme S2.** Preparation of disulfide bridged AS1411-PTX conjugates. Note for reagents and conditions: (i) I_2_, MeOH, TEA; (ii) AcCl, 65 °C reflux; (iii) PTX, Py; (iv) Amino-DNA, PB buffer (pH=8.0), DMF. Compound 9 indicated ASSP.

**Scheme S3.** Preparation of thioketal bridged AS1411-PTX conjugates. Note for reagents and conditions: (i) H_2_SO_4_, acetone; (ii) PTX, DCC, DMAP, DMF; (iii) Amino-DNA, DCC, HBTU, DIPEA, H_2_O, DMF. Compound 12 indicated ATKP.


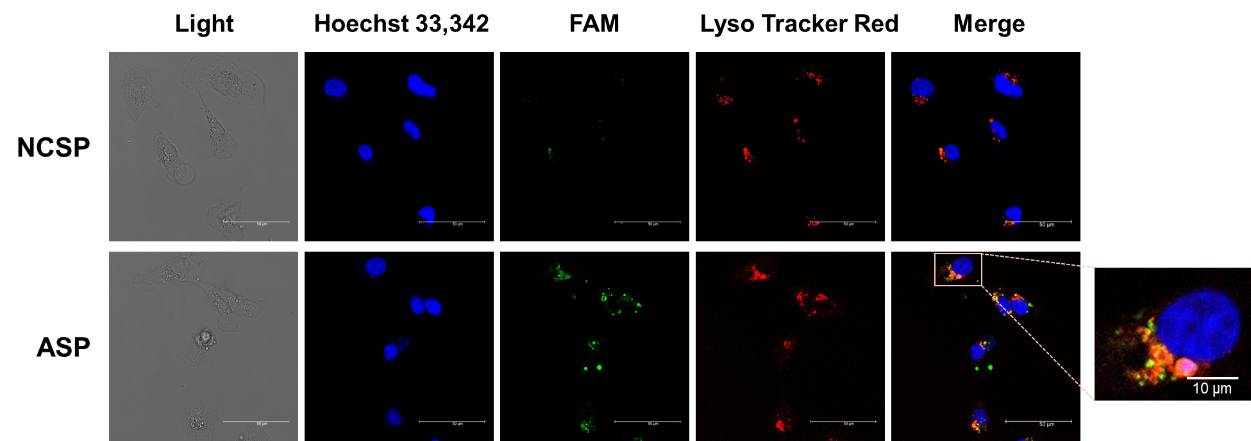


**Figure S1**. Representative images showing the co-localization of the conjugated PTX (Green) with a lysosomal marker (Lyso-Tracker Red; red) by confocal microscopy. The nuclei were counterstained with Hoechst 33,342 (blue). Scale bar, 50 μm.


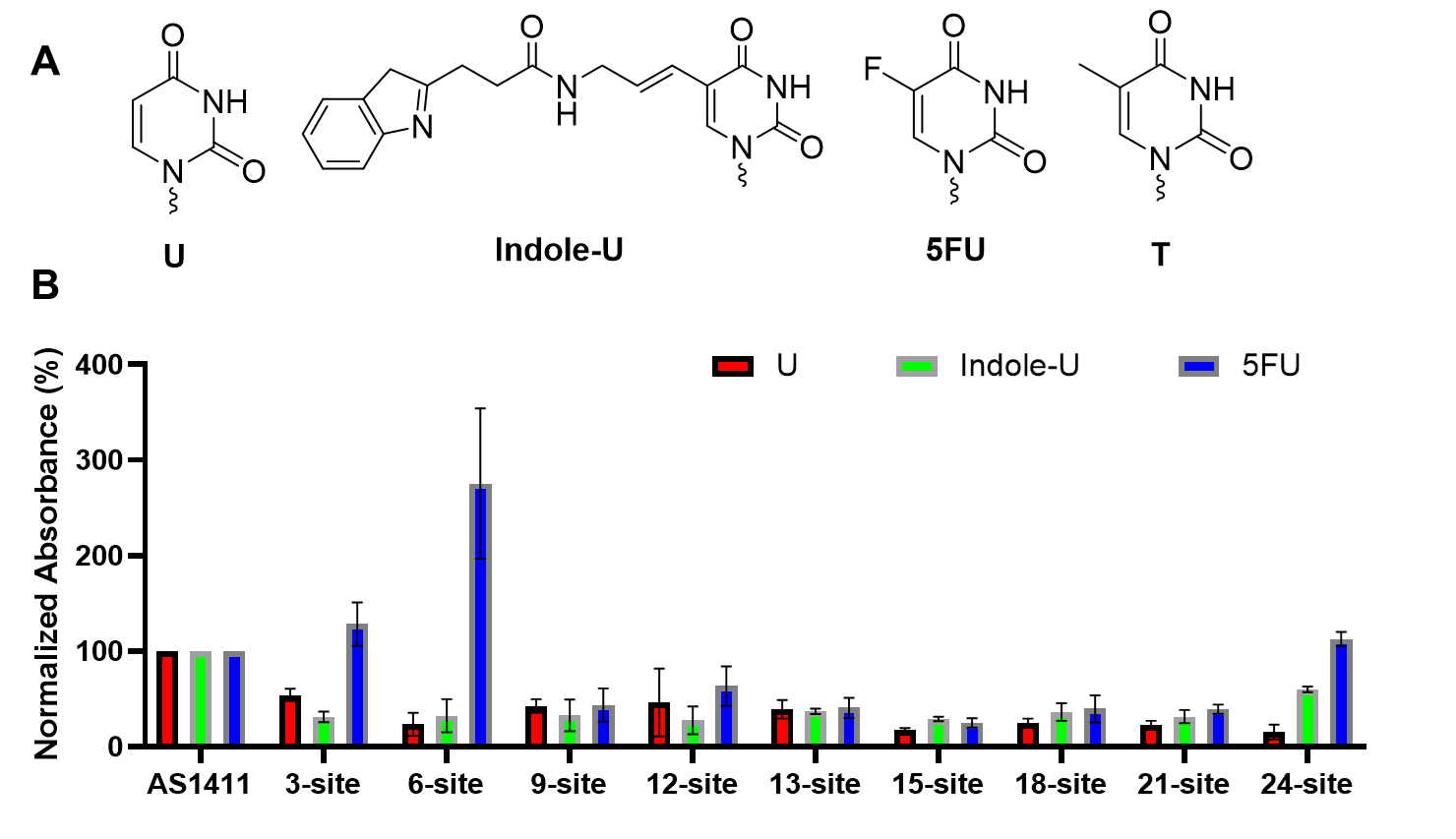


**Figure S2.** (**A**) The chemical structures used in aptamers. U represented uridine. Indole-U represented 5-indole-uridine. 5FU represented 5-fluoro-uridine. T represented naturally unmodified thymidine. (**B**) The binding ability of the 5FU modified AS1411s to nucleolin. The X-axis ordered in the modified sites (counting from 5'-terminus). The Y-axis was the normalized absorbance at 450 nm, which represented the binding ability of the modified AS1411s to nucleolin protein. The absorbance of unmodified AS1411 was treated as 100%. The data were presented as the means ± standard deviation from at least three replicates.


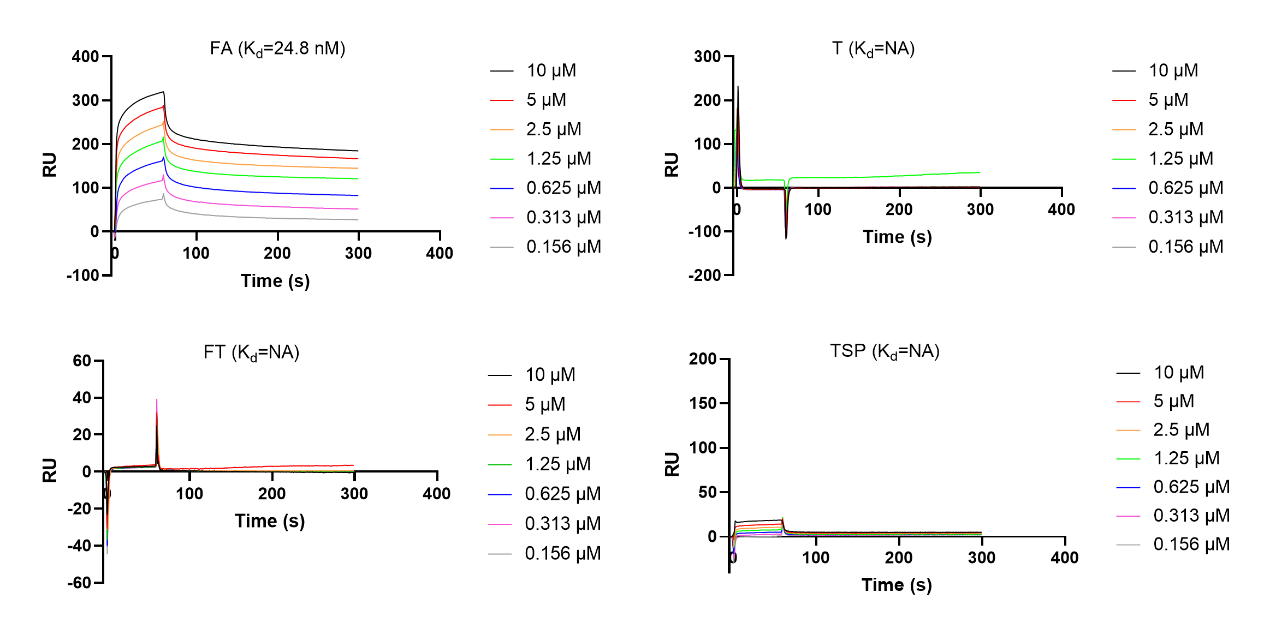


**Figure S3.** The binding affinity of modified aptamers to nucleolin. FA represented c modified AS1411 at site 6. T represented T-riched oligomer (negative control sequence). FT represented 5FU modified T-riched oligomer at site 6. TSP represented T-riched oligomer-paclitaxel conjugate with thioether linker. RU represented resonance units. NA represented that the calculated K_d_ value was not available. The concentrations of the ApDCs and aptamers ranged from 0.156 µM to 10 µM.


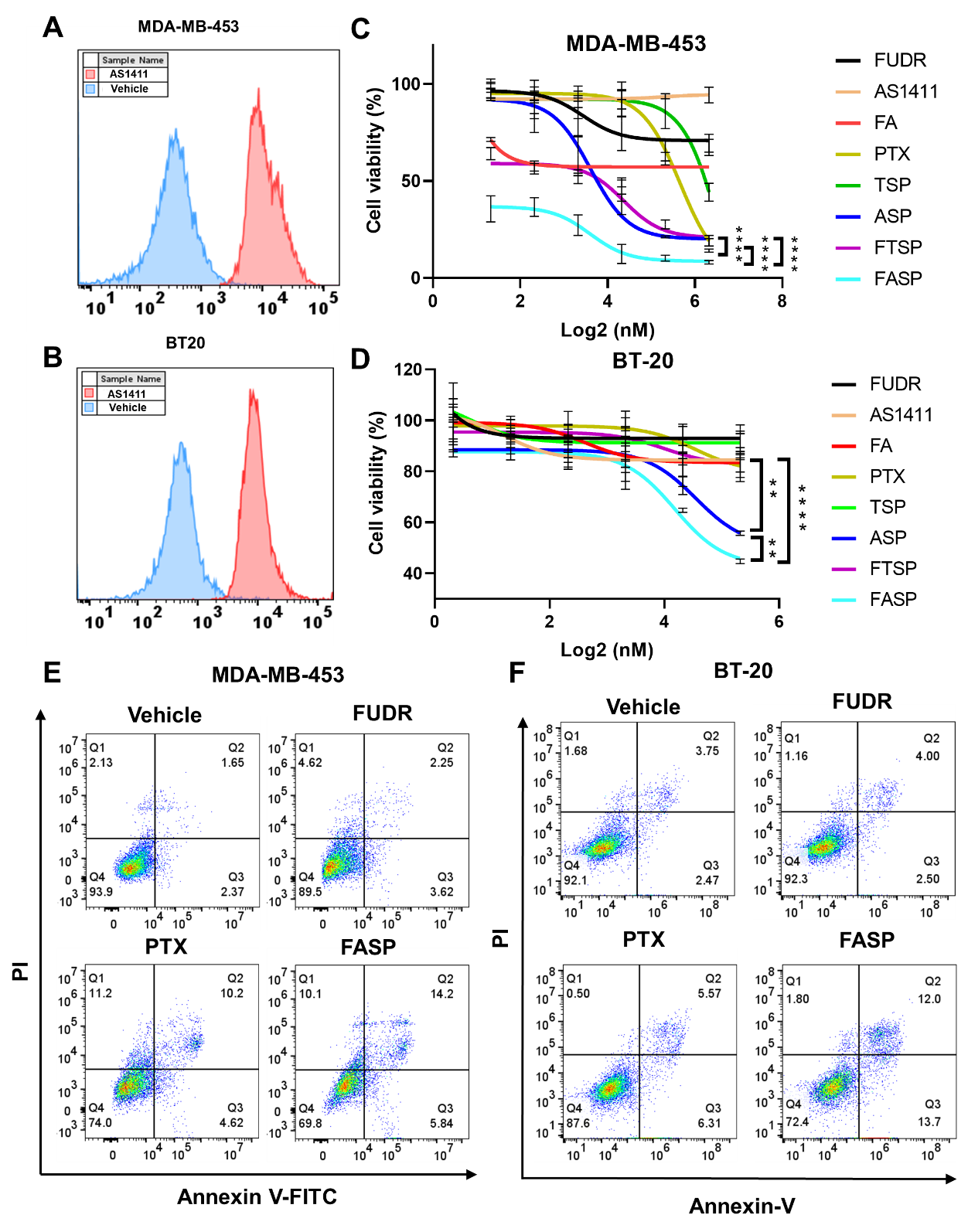


**Figure S4**. The anticancer effects of fluorouracil modified AS1411-paclitaxel conjugate with thioether linker on MDA-Mb-231 cells and on BT-20 cells. The targeting ability of AS1411 to MDA-MB-453 cells (**A**) and BT-20 cells (**B**) evaluated by flow cytometry using APC channel. The concentration of each sample was 4 nM. Anti-proliferation effect of FUDR, AS1411, FA, PTX, TSP, ASP, FTSP and FASP on MDA-MB-453 cells (**C**) and BT-20 cells (**D**) evaluated by CCK8 assay. The concentrations of each sample ranged from 1.25 nM to 80 nM. Data were expressed as mean ± standard deviation from at least three replicates, followed by two-way ANOVA with Sidak test. ** P < 0.01; **** P < 0.0001.

**Table S1**. MS-identification of aptamer-drug conjugates

| Name | Chemical Formula | Molecular  Weight | Exact Mass  [M] | Found  [M] |
| --- | --- | --- | --- | --- |
| ASP | C_318_H_391_N_105_O_183_P_26_S | 9449.5 | 9444.7 | 9446.6 |
| ASSP | C_320_H_395_N_105_O_183_P_26_S_2_ | 9509.6 | 9504.7 | 9507.0 |
| ATKP | C_323_H_401_N_105_O_183_P_26_S_2_ | 9551.7 | 9546.8 | 9547.5 |
| TSP | C_318_H_408_N_54_O_200_P_26_S | 9024.3 | 9019.6 | 9017.8 |
| FTSP | C_317_H_405_N_54_O_200_P_26_SF | 9028.2 | 9023.6 | 9023.3 |
| FASP | C_317_H_388_N_105_O_183_P_26_SF | 9453.4 | 9448.7 | 9448.4 |
| Cy5-ASP | C_357_H_444_N_108_O_187_P_27_S | 10108.3 | 10103.1 | 10100.9 |
| Cy5-FASP | C_356_H_441_N_108_O_187_P_27_SF | 10112.3 | 10107.1 | 10104.4 |

**Table S2**. The sequences of chemically modified aptamer used in the study

| Name | Sequence (5’-3’) |
| --- | --- |
| AS1411 | GGT GGT GGT GGT TGT GGT GGT GGT GG |
| 3-site | GGX GGT GGT GGT TGT GGT GGT GGT GG |
| 6-site | GGT GGX GGT GGT TGT GGT GGT GGT GG |
| 9-site | GGT GGT GGX GGT TGT GGT GGT GGT GG |
| 12-site | GGT GGT GGT GGX TGT GGT GGT GGT GG |
| 13-site | GGT GGT GGT GGT XGT GGT GGT GGT GG |
| 15-site | GGT GGT GGT GGT TGX GGT GGT GGT GG |
| 18-site | GGT GGT GGT GGT TGT GGX GGT GGT GG |
| 21-site | GGT GGT GGT GGT TGT GGT GGX GGT GG |
| 24-site | GGT GGT GGT GGT TGT GGT GGT GGX GG |
| 1 | GGF GGT GGT GGT TGT GGT GGT GGT GG |
| 2 | GGT GGF GGT GGT TGT GGT GGT GGT GG |
| 3 | GGT GGT GGF GGT TGT GGT GGT GGT GG |
| 4 | GGT GGT GGT GGT TGT GGF GGT GGT GG |
| 5 | GGT GGT GGT GGT TGT GGT GGT GGF GG |
| 6 | GGF GGF GGT GGT TGT GGT GGT GGT GG |
| 7 | GGF GGT GGF GGT TGT GGT GGT GGT GG |
| 8 | GGF GGT GGT GGT TGT GGF GGT GGT GG |
| 9 | GGF GGT GGT GGT TGT GGT GGT GGF GG |
| 10 | GGT GGF GGF GGT TGT GGT GGT GGT GG |
| 11 | GGT GGF GGT GGT TGT GGF GGT GGT GG |
| 12 | GGT GGF GGT GGT TGT GGT GGT GGF GG |
| 13 | GGT GGT GGF GGT TGT GGF GGT GGT GG |
| 14 | GGT GGT GGF GGT TGT GGT GGT GGF GG |
| 15 | GGT GGT GGT GGT TGT GGF GGT GGF GG |
| 16 | GGF GGF GGF GGT TGT GGT GGT GGT GG |
| 17 | GGF GGF GGT GGT TGT GGF GGT GGT GG |
| 18 | GGF GGF GGT GGT TGT GGT GGT GGF GG |
| 19 | GGF GGT GGF GGT TGT GGF GGT GGT GG |
| 20 | GGF GGT GGF GGT TGT GGT GGT GGF GG |
| 21 | GGF GGT GGT GGT TGT GGF GGT GGF GG |
| 22 | GGT GGF GGF GGT TGT GGF GGT GGT GG |
| 23 | GGT GGF GGF GGT TGT GGT GGT GGF GG |
| 24 | GGT GGF GGT GGT TGT GGF GGT GGF GG |
| 25 | GGT GGT GGF GGT TGT GGF GGT GGF GG |
| 26 | GGF GGF GGF GGT TGT GGF GGT GGT GG |
| 27 | GGF GGF GGF GGT TGT GGT GGT GGF GG |
| 28 | GGF GGF GGT GGT TGT GGF GGT GGF GG |
| 29 | GGF GGT GGF GGT TGT GGF GGT GGF GG |
| 30 | GGT GGF GGF GGT TGT GGF GGT GGF GG |
| 31 | GGF GGF GGF GGT TGT GGF GGT GGF GG |

**Note:** T represented naturally unmodified 2'-deoxythymidine; G represented naturally unmodified 2'- deoxyguanosine; X represents either 5-indole-2'-deoxyuridine (Indole-dU), fluorouracil (5FU) or 2'-deoxyuridine (dU); F represents fluorouracil (5FU)

**Table S3.** The predicted interaction of AS1411 and FA with NCL RBD1/2

| **Aptamer sites** | **Interaction**  **(AS1411-2KRR)** | **Interaction**  **(FA-2KRR)** |
| --- | --- | --- |
| T6 | LYS49 S | LYS49 S |
| G7 | LYS49 S; LYS78 S;  ARG121 P | LYS49 S; LYS78 S;  ARG121 P*2 |
| G8 | LYS88 S | LYS88 S |
| T9 | LYS88 S; ARG91 H;  THR95 H; ARG121 H*2 | LYS88 S; ARG91 H;  THR95 H; ARG121 H*2 |
| G10 | GLU119 S | GLU119 S |
| T12 | LYS138 P | LYS138 P |
| G17 | ARG91 S | ARG91 S |
| G20 | ASN14 H | ASN14 H; LYS78 S |
| Number of the interactions | 14 | 16 |
| Binding energy (kcal/mol) | -1494.0249 | -1564.6336 |

Notes: H, Hydrogen bond;P, Pi cation; S, Salt bridge

**2. Experimental Section**

**Synthesis of compound 2:** To a mixture suspension of 1,4-oxathiane-2,6-dione (397 mg, 3.006 mmol) in CH_2_Cl_2_ (5 mL), Py was added (242 μL, 3.006 mmol) and stirred for 5 min. Then, addition of PTX (2.176 g, 2.548 mmol) in anhydrous CH_2_Cl_2_ (20 mL) was performed and stirred at room temperature for 4 days. The mixture was concentrated under reduced pressure, following co-evaporated with toluene three times to remove the Py. Thereafter, the residue was chromatographed on silica gel (PE/EA=1/1) to give the compound 2 (1.807 g, 72%) as a white solid. 2-((2-(((1S,2R)-1-benzamido-3-(((2aR,4S,4aS,6R,9S,11S,12S,12bS)-6,12b-diacetoxy-12-(benzoyloxy)-4,11-dihydroxy-4a,8,13,13-tetramethyl-5-oxo-2a,3,4,4a,5,6,9,10,11,12,12a,12b-dodecahydro-1H-7,11-methanocyclodeca[3,4]benzo[1,2-b]oxet-9-yl)oxy)-3-oxo-1-phenylpropan-2-yl)oxy)-2-oxoethyl)thio)acetic acid: MS (ESI) m/z for C_51_H_54_NO_17_S^-^ [M-H]^-^, Calculated 984.3118, Found 984.3153; ^1^H NMR (400 MHz, MeOD) δ 8.02 (dd, *J* = 8.2, 6.8 Hz, 2H), 7.74 (dd, *J* = 5.3, 3.2 Hz, 2H), 7.60 – 7.55 (m, 1H), 7.49 (t, *J* = 7.5 Hz, 2H), 7.44 – 7.38 (m, 3H), 7.37 – 7.31 (m, 4H), 7.17 (t, *J* = 7.3 Hz, 1H), 6.35 (d, *J* = 4.5 Hz, 1H), 5.99 (t, *J* = 8.6 Hz, 1H), 5.77 (d, *J* = 6.1 Hz, 1H), 5.54 (d, *J* = 7.3 Hz, 1H), 5.41 (d, *J* = 6.1 Hz, 1H), 4.93 – 4.87 (m, 1H), 4.25 (dd, *J* = 11.0, 6.7 Hz, 1H), 4.09 (s, 2H), 3.72 (d, *J* = 7.2 Hz, 1H), 3.56 – 3.35 (m, 3H), 3.30 – 3.24 (m, 1H), 3.16 (d, *J* = 15.0 Hz, 1H), 2.43 – 2.24 (m, 4H), 2.15 – 2.03 (m, 4H), 1.85 – 1.65 (m, 5H), 1.55 (s, 3H), 1.04 (d, *J* = 3.5 Hz, 6H); ^13^C NMR (101 MHz, MeOD) δ 205.24, 171.33, 170.24, 167.71, 142.40, 138.37, 135.48, 134.90, 134.66, 132.93, 131.34, 130.14, 129.97 – 129.47, 128.87 – 128.4, 85.93, 82.28, 79.04, 77.49, 76.85, 76.51, 76.28, 73.07, 72.33, 59.24, 55.24, 47.92, 44.62, 37.55, 36.45, 33.68, 26.97, 23.35, 22.43, 20.84, 14.99, 10.50. Peak overlapping was observed.

**Synthesis of compound 3:** To a mixture solution of compound 2 (1.2 g, 1.218 mmol) and NHS (147 mg, 1.279 mmol) in anhydrous THF (30 mL), DCC (276 mg, 1.34 mmol) was added and stirred at room temperature for 3 days. The mixture was filtered, and the filtrate was concentrated in acuum to give the crude solid. Finally, the residue was recrystallized from diethyl ether (100 mL) being cooled to 4 °C, affording white solid (1.18 g) after evaporated to dryness. No further operation was performed. 2aR,4S,4aS,6R,9S,11S,12S,12bS)-9-(((2R,3S)-3-benzamido-2-(2-((2-((2,5-dioxopyrrolidin-1-yl)oxy)-2-oxoethyl)thio)acetoxy)-3-phenylpropanoyl)oxy)-12-(benzoyloxy)-4,11-dihydroxy-4a,8,13,13-tetramethyl-5-oxo-3,4,4a,5,6,9,10,11,12,12a-decahydro-1H-7,11-methanocyclodeca[3,4]benzo[1,2-b]oxete-6,12b(2aH)-diyl diacetate: MS (ESI) m/z for C_55_H_57_N_2_O_19_S^-^ [M-H]^-^, Calculated 1081.3282, Found 1081.3203.

**Synthesis of compound 4:** To a solution of 3′-amino DNA (100 nmol) in NaHCO_3_ buffer (50 mM, 0.1 mL), compound 3 (3 µmol) in DMF (0.2 mL) was added to the solution and stirred (800 rpm) for 2 h at 30 °C. The reaction mixture was quenched by the addition of the TEAA (2 M, 0.1 mL). The solvent was removed in vacuo, and the solid residue was re-dissolved in ddH_2_O (1 mL). Afterwards, the mixture was purified using high-performance liquid chromatography (Agilent 1260). Phase A was CAN, and phase B was TEAA (50 mM). The gradient was run from 5% to 60% of phase A in 30 minutes for the Xbride ®Oligonucleotide BEH C18 OBDTM Prep Column (2.5 μm, 10 mm×50 mm) at a flow rate of 1.2 mL·min^-1^ with ambient column temperature. The purified ApDCs were desalted by using a Sephadex G25 column to afford compound 4/5. The samples were further lyophilized to dryness for storage, and confirmed by ESI MS analyses.

**Synthesis of compound 6:** To a solution of compound 5 (2.3 g, 21.7 mmol) in methanol (30 mL) being cooled to 0 °C, Iodine (5.634 g, 11.1 mmol) in methanol (30 mL) was added dropwise to the stirred solution for 2 hours. TLC analysis showed the complete consumption of compound 6. The reaction mixture was quenched by the addition of the saturated aqueous Na_2_S_2_O_3_, and the pH was adjusted to around 4 by progressively adding solid NaHCO_3_. Afterwards, the mixture was filtered and concentrated, chromatographed on silica gel (DCM/MeOH=30/1) to give the compound 7 (2.012 g, 87%) as a white solid. 3,3’-disulfanediyldipropionic acid: MS (ESI) m/z for C_6_H_9_O_4_S_2_^-^ [M-H]^-^, Calculated 208.9948, Found 208.9964; ^1^H NMR (400 MHz, MeOD) δ 2.92 (t, *J* = 7.0 Hz, 4H), 2.71 (t, *J* = 7.0 Hz, 4H). ^13^C NMR (101 MHz, MeOD) δ 175.36, 34.76, 34.30.

**Synthesis of compound 8:** A round-bottom flask was charged with compound 6 (0.211 g, 1.005 mmol) and [acetylchloride](C:/Program%20Files%20(x86)/Youdao/Dict/8.9.8.0/resultui/html/index.html#/javascript:;) (5 mL). Then, the mixture under nitrogen was heated to 65 °C under reflux for 2 hours, to afford crude compound 7. No further purification was performed. 1,5,6-oxadithionane-2,9-dione: MS (ESI) m/z for C_6_H_9_O_3_S_2_^+^ [M+H]^+^, Calculated 192.9988, Found 192.9990.

All crude compound 7 and PTX (854 mg, 1 mmol) were dissolved in anhydrous DCM (20 mL). Then, Py (0.282 mL, 3.5 mmol) was added dropwise to the mixture, and stirred at room temperature for 2 days. The solvent was removed in vacuo, and the solid residue was re-dissolved in DCM (20 mL). Afterwards, the residue was chromatographed on silica gel (DCM/MeOH=20/1~3/1) to yield the compound 9 (648 mg, 62%) as a white solid. 3-((3-(((1S,2R)-1-benzamido-3-(((2aR,4S,4aS,6R,9S,11S,12S,12bS)-6,12b-diacetoxy-12-(benzoyloxy)-4,11-dihydroxy-4a,8,13,13-tetramethyl-5-oxo-2a,3,4,4a,5,6,9,10,11,12,12a,12b-dodecahydro-1H-7,11-methanocyclodeca[3,4]benzo[1,2-b]oxet-9-yl)oxy)-3-oxo-1-phenylpropan-2-yl)oxy)-3-oxopropyl)disulfanyl)propanoic acid: MS (ESI) m/z for C_53_H_58_NO_17_S_2_^-^ [M-H]^-^, Calculated 1044.3152, Found 1044.3238; ^1^H NMR (400 MHz, MeOD) δ 8.02 (dd, *J* = 8.2, 6.8 Hz, 2H), 7.74 (dd, *J* = 5.3, 3.2 Hz, 2H), 7.60 – 7.55 (m, 1H), 7.49 (t, *J* = 7.5 Hz, 2H), 7.44 – 7.38 (m, 3H), 7.37 – 7.31 (m, 4H), 7.17 (t, *J* = 7.3 Hz, 1H), 6.35 (d, *J* = 4.5 Hz, 1H), 5.99 (t, *J* = 8.6 Hz, 1H), 5.77 (d, *J* = 6.1 Hz, 1H), 5.54 (d, *J* = 7.3 Hz, 1H), 5.41 (d, *J* = 6.1 Hz, 1H), 4.93 – 4.87 (m, 1H), 4.25 (dd, *J* = 11.0, 6.7 Hz, 1H), 4.09 (s, 2H), 3.72 (d, *J* = 7.2 Hz, 1H), 3.56 – 3.35 (m, 3H), 3.30 – 3.24 (m, 1H), 3.16 (d, *J* = 15.0 Hz, 1H), 2.43 – 2.24 (m, 4H), 2.15 – 2.03 (m, 4H), 1.85 – 1.65 (m, 5H), 1.55 (s, 3H), 1.04 (d, *J* = 3.5 Hz, 6H); ^13^C NMR (101 MHz, MeOD) δ 205.19, 172.60, 171.63, 171.31, 170.61, 170.34, 167.66, 142.38, 138.29, 135.54, 134.87, 134.64, 132.95, 131.32, 130.14, 129.68, 128.67, 85.92, 82.28, 79.03, 77.47, 76.84, 76.16, 73.02, 72.33, 59.23, 55.24, 49.90, 47.93, 44.61, 37.56, 36.43, 35.58, 34.78, 34.47, 33.71, 26.96, 23.35, 22.43, 20.85, 15.02, 10.51. Peak overlapping was observed.

**Synthesis of compound 9:** To a solution of 3′-amino DNA (100 nmol) in PB buffer (50 mM, 0.1 mL), compound 10 (3 µmol) in DMF (0.2 mL) was added to the solution and stirred (800 rpm) for 2 h at 30 °C. The reaction mixture was quenched by the addition of the TEAA (2M, 0.1 mL). The solvent was removed in vacuo, and the solid residue was re-dissolved in ddH_2_O (1 mL). Afterwards, the mixture was purified using high-performance liquid chromatography (Agilent 1260). Phase A was CAN, and phase B was TEAA (50 mM). The gradient was run from 5% to 60% of phase A in 30 minutes for the Xbride ®Oligonucleotide BEH C18 OBDTM Prep Column (2.5 μm, 10 mm×50 mm) at a flow rate of 1.2 mL·min^-1^ with ambient column temperature. The purified ApDCs were desalted by using a Sephadex G25 column to afford compound 11/12. The samples were further lyophilized to dryness for storage, and confirmed by ESI MS analyses.

**Synthesis of compound 10:** To a solution of compound 5 (3.412 g, 32.2 mmol) in acetone (20 mL) being cooled to 0 °C, [sulfuric](C:/Program%20Files%20(x86)/Youdao/Dict/8.9.8.0/resultui/html/index.html#/javascript:;) [acid](C:/Program%20Files%20(x86)/Youdao/Dict/8.9.8.0/resultui/html/index.html#/javascript:;) (1.882 g, 19.2 mmol) was added dropwise to the stirred mixture for 2 hours. Then, the mixture was diluted with ddH_2_O (200 mL), and the pH was adjusted to 5 by progressively adding aqueous NaOH. Thereafter, the mixture was evaporated to remove the acetone, and the aqueous layer was extracted with ethyl acetate (100 mL×3). Finally, the crude product was chromatographed on silica gel ([n-hexane](C:/Program%20Files%20(x86)/Youdao/Dict/8.9.8.0/resultui/html/index.html#/javascript:;)/EA=4/1) under reduced pressure to yield the compound 13 (2.313 g, 57%) as a white solid. 3,3’-(propane-2,2-diylbis(sulfanediyl))dipropionic acid: MS (ESI) m/z for C_9_H_15_O_4_S_2_^-^ [M-H]^-^, Calculated 251.0417, Found 251.0448; ^1^H NMR (400 MHz, MeOD) δ 2.76 (dd, *J* = 9.0, 5.4 Hz, 4H), 2.49 (t, *J* = 7.1 Hz, 4H), 1.49 (s, 6H); ^13^C NMR (101 MHz, MeOD) δ 56.97, 35.33, 31.31, 26.33.

**Synthesis of compound 11:** A round-bottom flask was charged with compound 10 (0.433 g, 1.718 mmol) and PTX (1.547 g, 1.812 mmol) in DMF (20 mL). DCC (355 mg, 1.722 mmol) and a catalytic amount of DMAP were added into the solution. The mixture was left standing overnight at room temperature. The mixture was filtered, evaporated, and chromatographed on silica gel (DCM/MeOH=50/1~30/1) to yield the compound 11 (1.064 g, 57%) as a white solid. (3S,4R)-4-((((2aR,4S,4aS,6R,9S,11S,12S,12bS)-6,12b-diacetoxy-12-(benzoyloxy)-4,11-dihydroxy-4a,8,13,13-tetramethyl-5-oxo-2a,3,4,4a,5,6,9,10,11,12,12a,12b-dodecahydro-1H-7,11-methanocyclodeca[3,4]benzo[1,2-b]oxet-9-yl)oxy)carbonyl)-10,10-dimethyl-1,6-dioxo-1,3-diphenyl-5-oxa-9,11-dithia-2-azatetradecan-14-oic acid: MS (ESI) m/z for C_56_H_64_NO_17_S_2_^-^ [M-H]^-^, Calculated 1086.3621, Found 1086.3747; ^1^H NMR (400 MHz, MeOD) δ 8.05 – 7.98 (m, 2H), 7.71 (dd, *J* = 5.2, 3.3 Hz, 2H), 7.57 (td, *J* = 7.0, 3.2 Hz, 1H), 7.39 (dddd, *J* = 23.6, 15.3, 11.1, 4.7 Hz, 9H), 7.17 (t, *J* = 7.3 Hz, 1H), 6.35 (s, 1H), 5.97 (t, *J* = 8.7 Hz, 1H), 5.77 (d, *J* = 6.5 Hz, 1H), 5.53 (d, *J* = 7.2 Hz, 1H), 5.43 (d, *J* = 6.5 Hz, 1H), 4.93 – 4.87 (m, 1H), 4.24 (dd, *J* = 10.9, 6.7 Hz, 1H), 4.09 (s, 2H), 3.71 (d, *J* = 7.2 Hz, 1H), 2.71 (t, *J* = 6.8 Hz, 2H), 2.61 (t, *J* = 6.6 Hz, 2H), 2.41 – 2.28 (m, 4H), 2.13 – 2.00 (m, 8H), 1.82 (d, *J* = 0.8 Hz, 3H), 1.77 – 1.65 (m, 2H), 1.55 (s, 3H), 1.34 (s, 3H), 1.24 – 1.07 (m, 3H), 1.06 – 0.99 (m, 6H); ^13^C NMR (101 MHz, MeOD) δ 205.19, 172.86, 171.66, 171.34, 170.41, 170.35, 167.68, 142.36, 138.36, 135.51, 134.94, 132.95, 131.44, 131.25, 130.16, 129.76, 129.60, 128.75, 128.72, 85.91, 82.29, 79.07, 76.82, 76.28, 76.03, 73.02, 31.19, 30.70, 26.15, 23.38, 22.41, 20.84, 15.08, 10.48. Peak overlapping was observed.

**Synthesis of compound 12:** The compound 11 (3 µmol), DCC (3 µmol) and HBTU (3 µmol) in DMF (0.4 mL) was added to the solution and stirred for 10 min. After being cooled to 0 °C, a solution of 5′/3′-amino DNA (100 nmol) and DIPEA (3 µmol) in ddH_2_O (0.1 mL) was added dropwise into the mixture and stirred (800 rpm) for 4 h at 30 °C. The reaction mixture was quenched by the addition of the TEAA (2 M, 0.1 mL). The solvent was removed in vacuo, and the solid residue was re-dissolved in ddH_2_O (1 mL). Afterwards, the mixture was purified using high-performance liquid chromatography (Agilent 1260). Phase A was ACN, and phase B was TEAA (50 mM). The gradient was run from 5% to 60% of phase A in 30 minutes for the Xbride ®Oligonucleotide BEH C18 OBDTM Prep Column (2.5 μm, 10 mm×50 mm) at a flow rate of 1.2 mL·min^-1^ with ambient column temperature. The purified ApDCs were desalted by using a Sephadex G25 column to afford compound 12. The samples were further lyophilized to dryness for storage, and confirmed by ESI MS analyses.

**FCM assays for cellular uptake**: MDA-MB-453 cells and BT-20 cells were seeded in a 10 cm dish at a density of 2.0×10^5^ cells and incubated overnight. The cells were washed three times with PBS and harvested with Accutase. Then, the cells were treated with Cy3-labeled AS141 at a concentration of 4 nM for 2 h, and analyzed using flow cytometry (FCM) in the PE channel.

**CCK-8 assay for cell viability:** Cell viability was assessed using the Cell Counting Kit-8 kit. Briefly, MDA-MB-453 cells, BT-20 cells were seeded in 96-well plates at a density of 5,000 cells per well and incubated overnight for adherence. Solutions of ApDCs and controls were prepared in medium, ranging in concentration from 1.25 nM to 80 nM. After removal of the cell culture medium, the solutions of ApDCs and controls were added, followed by a 72-hour incubation at 37 °C. After the incubation period, 100 µL of culture medium containing 10% CCK-8 solution was added to each well. The plates were further incubated for 2 hours and then read at 450 nm using a microplate reader.

**Cellular apoptosis assays**: Cellular apoptosis was assessed using the Annexin V-FITC/PI Apoptosis Kit. Briefly, MDA-MB-453 cells and BT-20 cells were seeded in 6-well plates at a density of 4×10^5^ cells per well and incubated overnight for adherence. Solutions of ApDCs and controls were prepared in medium at 200 nM. After removal of the cell culture medium, the solutions of ApDCs and controls were added, followed by a 48-hour incubation at 37 °C. After the incubation period, cells were harvested, rinsed with cold PBS, and resuspended in 200 μL 1 × Annexin-V binding buffer. Next, 5 μL Annexin-V–FITC (fluorescein isothiocyanate) and 10 μL PI (propidium iodide) were added to the cell suspension and incubated at room temperature for 15 min in the dark with gentle vertexing. Quantitative determination was performed using a flow cytometer. By analyzing the fluorescence signals obtained from the flow cytometer, we were able to assess the proportion of cells undergoing early apoptosis (Annexin-V positive, PI negative), late apoptosis (Annexin-V positive, PI positive), and necrosis (PI positive) in response to the treatment with ApDCs and respective controls.

**Attachment Figures**


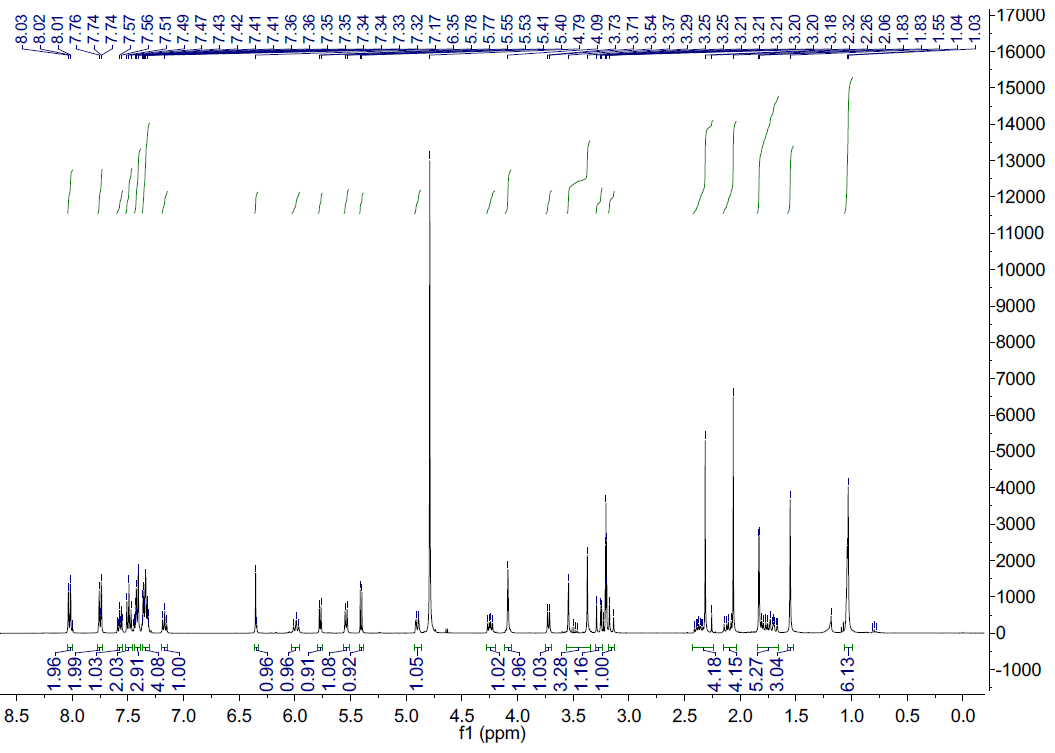


^1^H-NMR spectra of compound 2


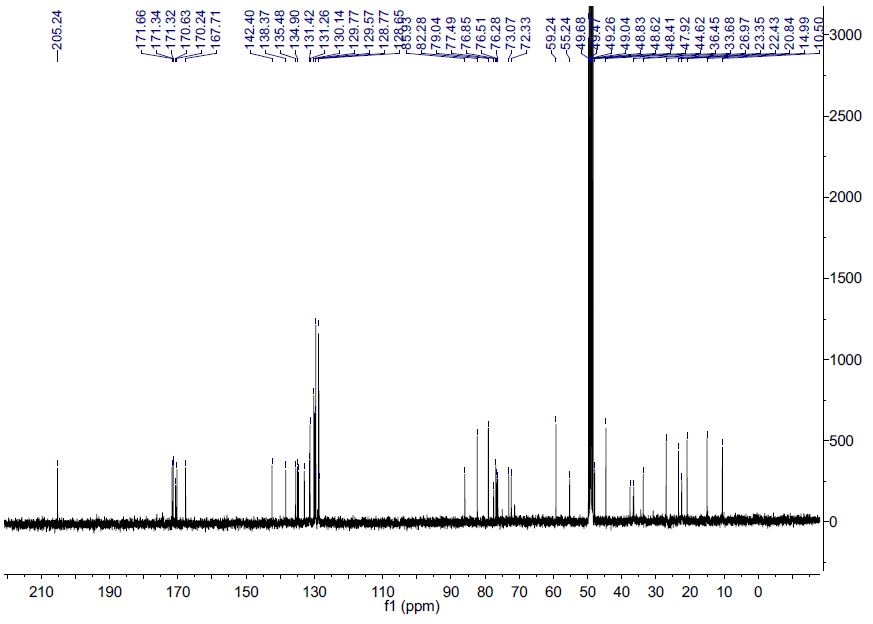


^13^C-NMR spectra of compound 2


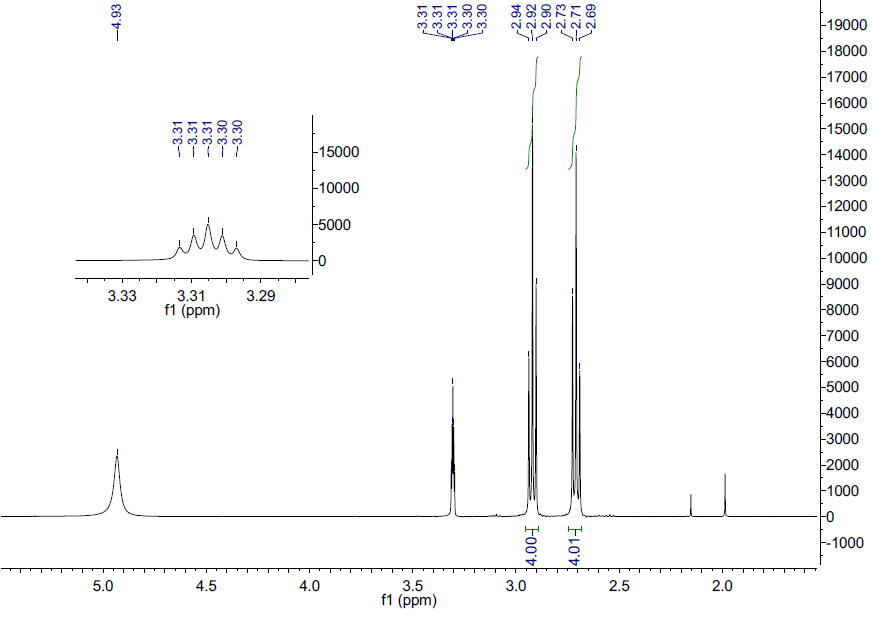


^1^H-NMR spectra of compound 6


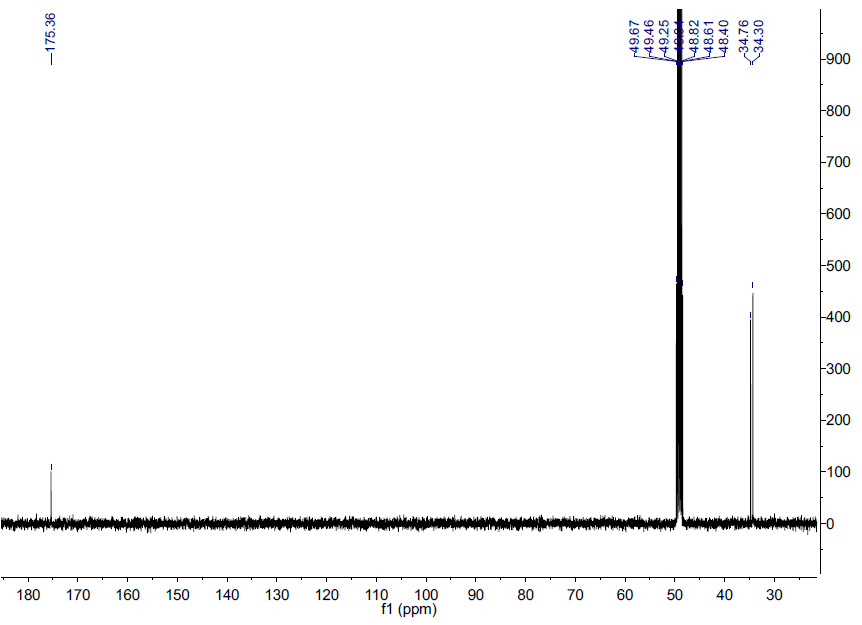


^13^C-NMR spectra of compound 6


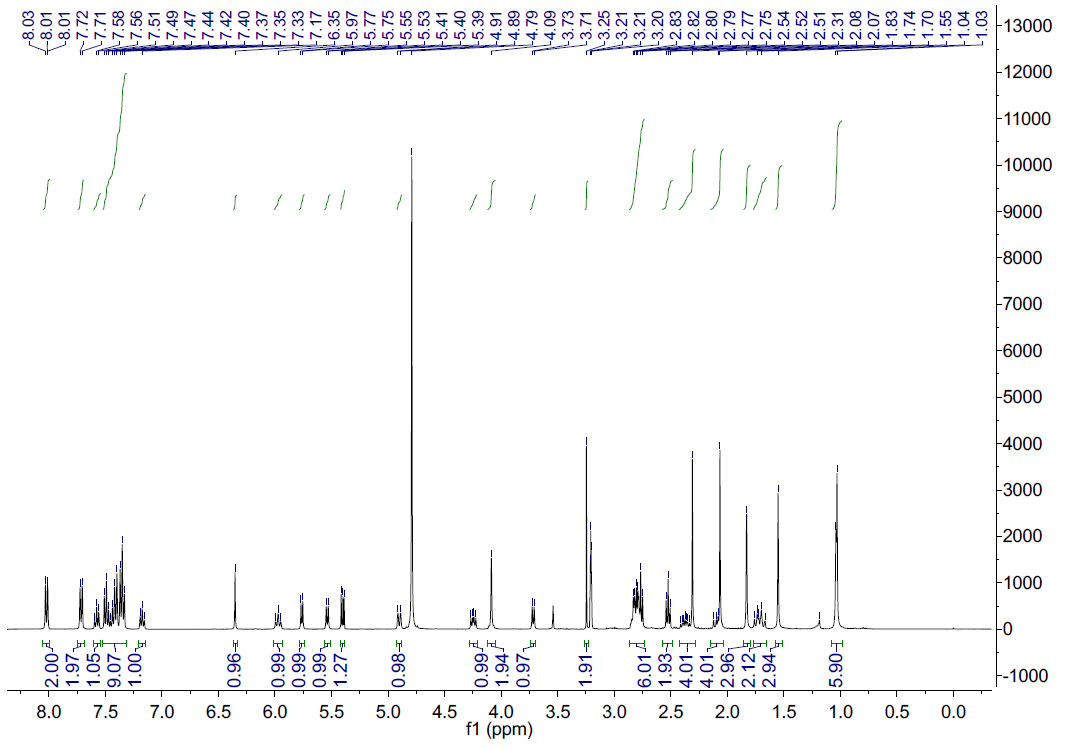


^1^H-NMR spectra of compound 8


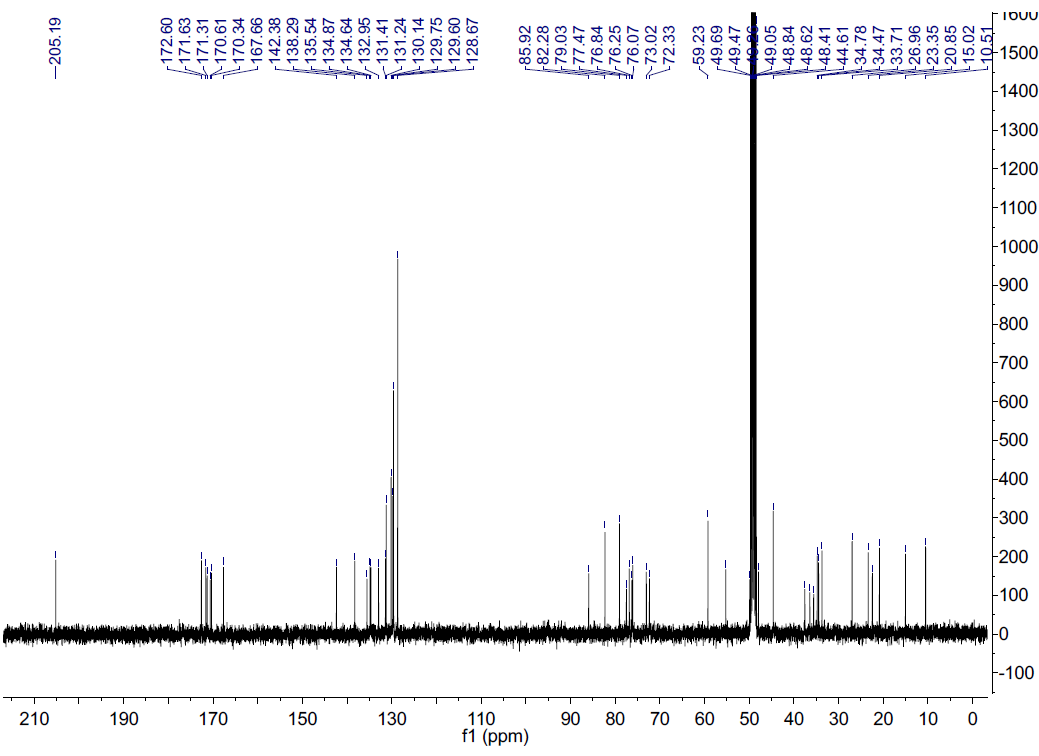


^13^C-NMR spectra of compound 8


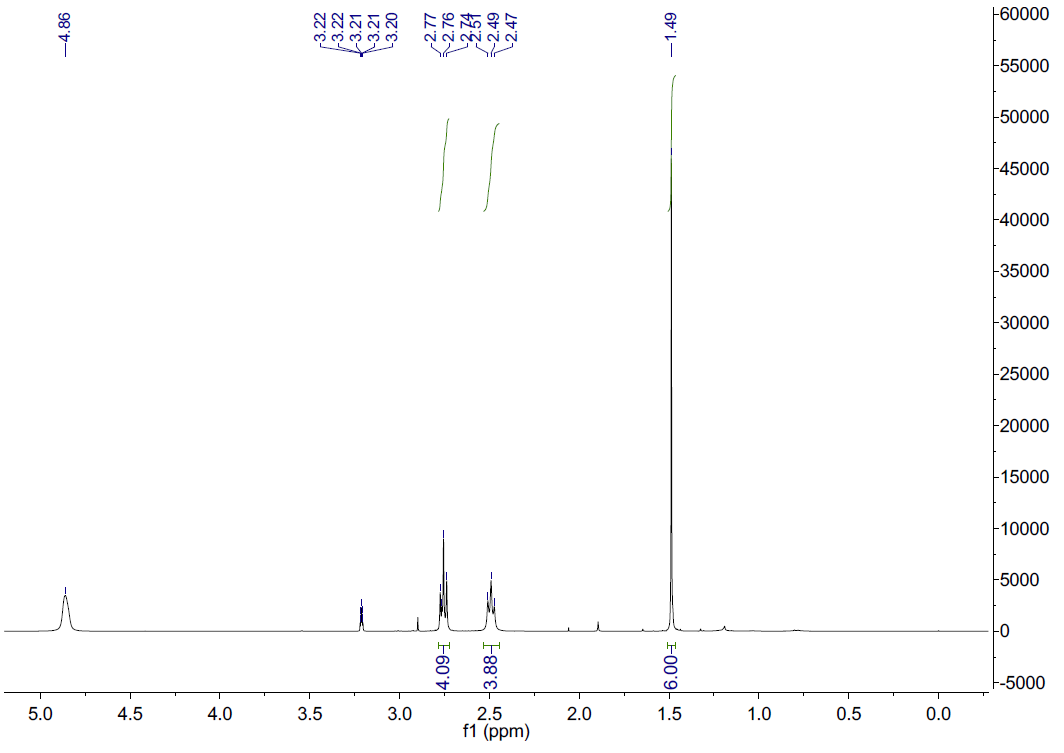


^1^H-NMR spectra of compound 10


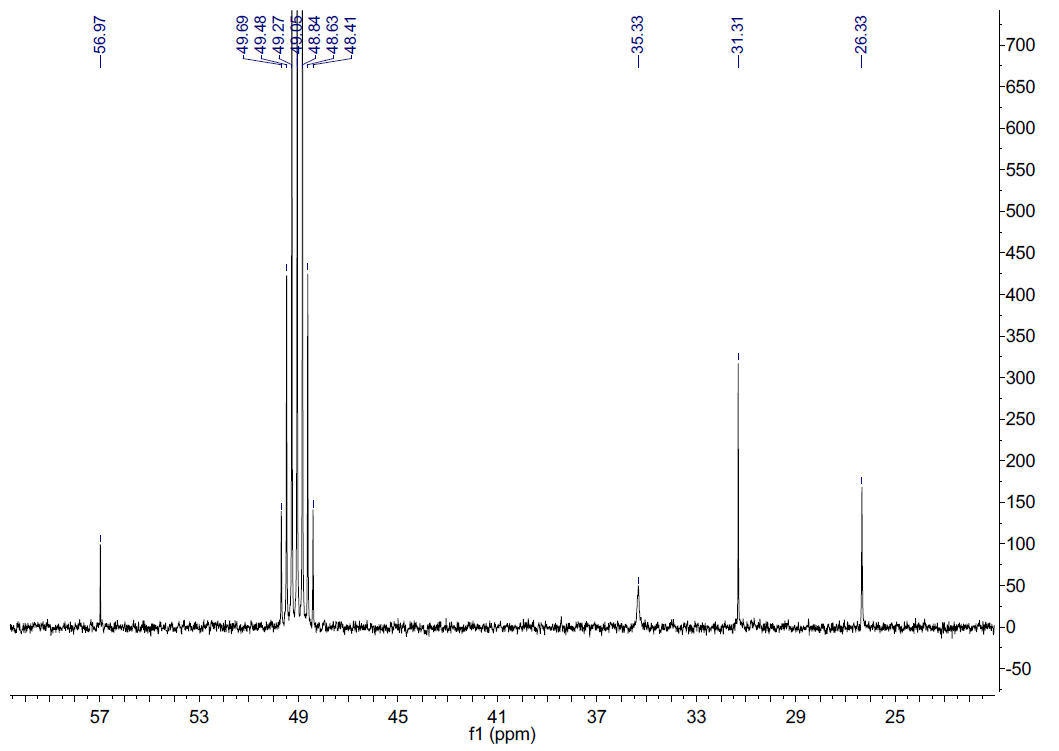


^13^C-NMR spectra of compound 10


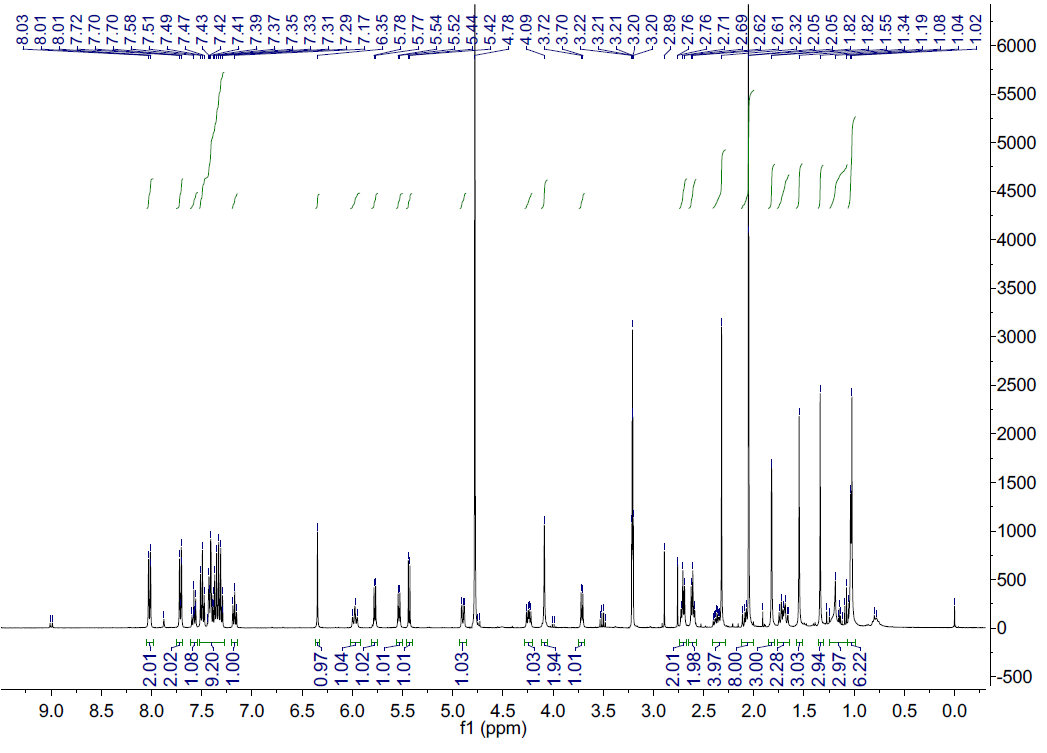


^1^H-NMR spectra of compound 11


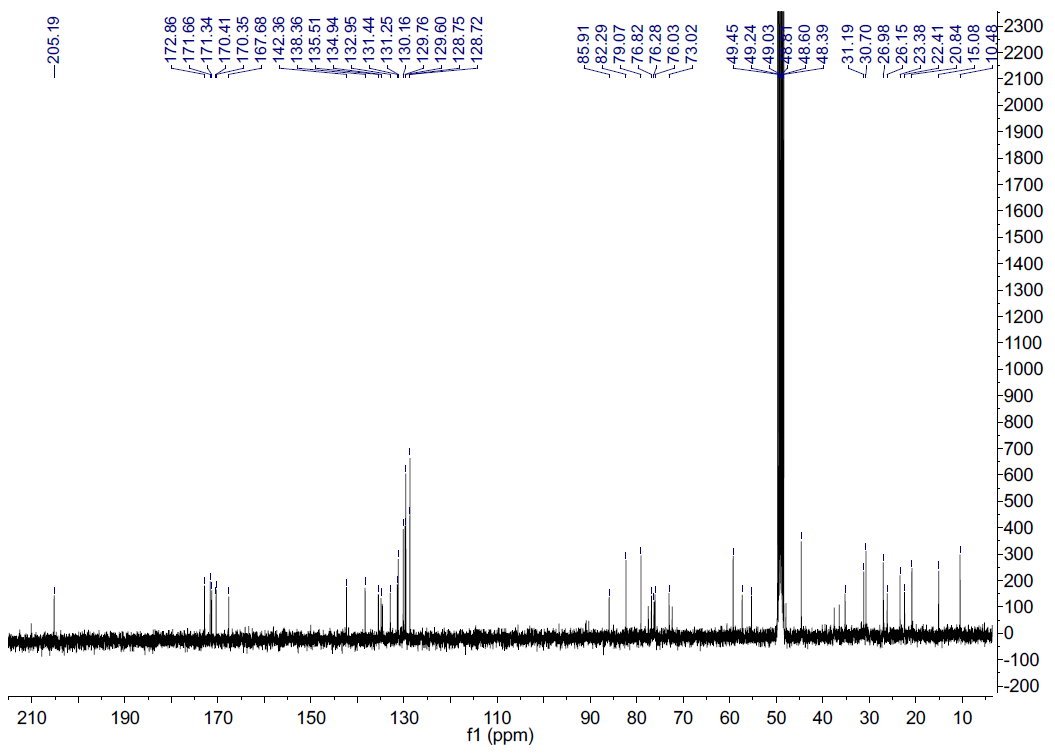


^13^C-NMR spectra of compound 11

MS spectra of ASP

MS spectra of ASSP

MS spectra of ATKP

MS spectra of TSP

MS spectra of ASP

MS spectra of FTSP

MS spectra of FASP

MS spectra of Cy5-ASP

MS spectra of Cy5-FASP
